# Progress of Gene‐Functionalized Regenerative Material Repair Intervertebral Disc Degeneration

**DOI:** 10.1002/smsc.202300355

**Published:** 2024-03-18

**Authors:** Xiaohu Li, Hongze Chang, Feng Cai, Yan Zhang, Ang Li, Xiaolong Yang, Zhengwei Cai, Wenguo Cui, Xiaodong Liu

**Affiliations:** ^1^ Department of Orthopedics Yangpu Hospital School of Medicine Tongji University Shanghai 200090 China; ^2^ Center for Clinical Research and Translational Medicine Yangpu Hospital School of Medicine Tongji University Shanghai 200090 China; ^3^ Department of Orthopaedics Shanghai Key Laboratory for Prevention and Treatment of Bone and Joint Diseases Shanghai Institute of Traumatology and Orthopaedics Ruijin Hospital Shanghai Jiao Tong University School of Medicine 197 Ruijin 2nd Road Shanghai 200025 P. R. China

**Keywords:** gene therapy, gene‐functionalized regenerative materials, intervertebral disc degeneration, regenerative materials

## Abstract

Intervertebral disc degeneration (IDD) is widely recognized as the primary culprit of chronic low back pain. Restoring deteriorated intervertebral disc (IVD) and alleviating IDD‐induced low back pain are remaining enormous challenges. There is a genetic susceptibility to IDD, and gene therapy has some therapeutic potential. However, traditional gene therapy still has certain drawbacks, including host immunity, temporary release, and suppression of gene medication function. Although regenerative materials can effectively improve the local microenvironment, they cannot treat IDD from the root. Gene‐functionalized regenerative material (GRM) is constructed based on physical embedding or forming chemical bonds by introducing particular genes, such as pDNA, siRNA, mRNA, and miRNA, into the regenerated materials. The findings demonstrate that GRM not only enhances the safety and controllability of gene therapy, but also effectively repairs IDD by overcoming the constraint that simple regenerative materials cannot reverse the disease's progression from the root. This article provided a brief overview of the physiological and pathological features of the IVD, genetic susceptibility to IDD, available treatment options, and their limitations. Then, the significance of GRM‐based treatment of IDD is proposed, and the future challenges and development in this field are finally prospected.

## Graphical abstract

Gene functionalized regenerative materials (GRM) have achieved significant progress in treating intervertebral disc degeneration (IDD). This review summarizes the physiological and pathological mechanisms, genetic susceptibility, and conventional treatments for IDD. It discusses the potential future of the field by focusing on the current advancements of various GRMs in IDD repair.

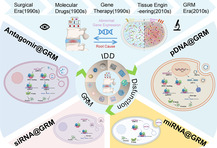

## 1 Introduction

Intervertebral discs (IVDs) are fibrous cartilage tissues that connect adjacent vertebrae, providing flexibility and stress relief for the spine. Many factors, such as genetics, smoking, obesity, abnormal stress, and increasing age, can cause intervertebral disc degeneration (IDD).^[^ ^1^ ^]^ Chronic low back pain caused by IDD is one of the top five musculoskeletal disorders that cause a significant economic burden worldwide.^[^ ^2^ ^]^ Currently, there is no practical way to treat IDD, let alone reverse the process of IDD from the root.

Traditional IDD therapy can relieve pain by conservative treatment or surgical resection, which has some drawbacks, including postoperative complications, treating symptoms rather than the underlying causes of pain, secondary operations, etc.^[^ ^3^ ^]^ Besides, it is also accompanied by a decrease in IVD cells and a severe deficiency of extracellular matrix (ECM) synthesis.^[^ ^4^ ^]^ Thus, local delivery of bioactive drugs such as small molecule drugs, peptides, and growth factors has shown specific potential in IVD repair by improving IDD's local microenvironment and increasing IVD cells’ bioactivity. However, there are issues with low drug activity and the need for repeated injections.^[^ ^5^ ^]^ Furthermore, it can increase cell transplant survival and promote IVD repair by preconditioning transplanted cells chemically or physically or delivering transplanted cells with regenerative materials.^[^ ^5^ ^]^ Nonetheless, the treatment of IDD is a lengthy process, and the growth and proliferation of IVD cells require long‐term microenvironment homeostasis. Consequently, pretreatment of transplanted cells or delivery of cells by regenerative materials alone cannot reverse the degenerative IVD microenvironment at the root; that is, it cannot provide an effective guarantee for the survival and proliferation of IVD cell.^[^ ^6^ ^]^ In conclusion, there are still many problems in repairing IDD based on regenerative materials loaded with bioactive drugs and stem cells, and the IDD process cannot be fundamentally reversed.

**Figure 1:**
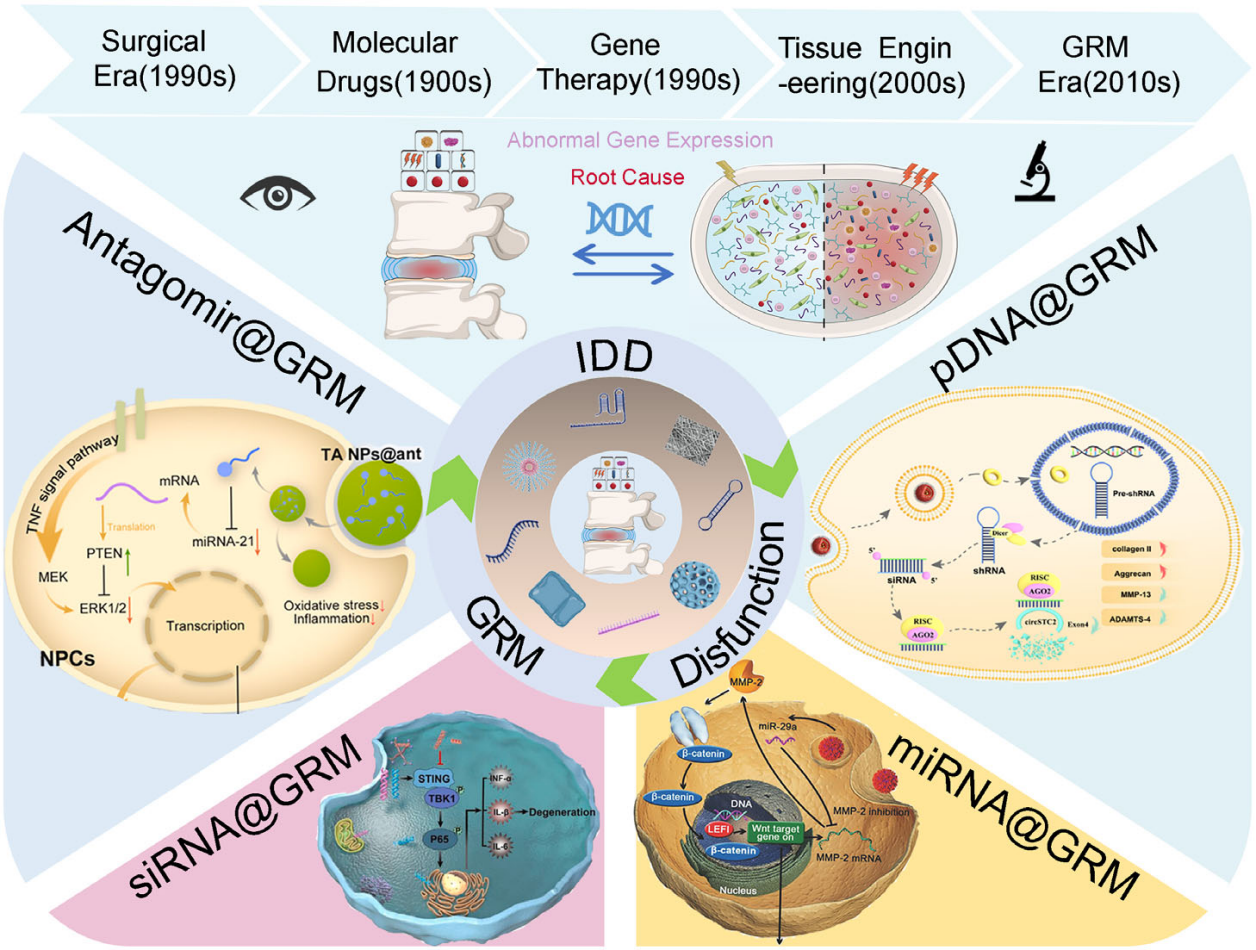
The mechanism and significance of GRM in repairing IDD and the development history of IDD treatment strategy are illustrated.

As the basic genetic unit of organisms, genes significantly impact the onset and progression of diseases.^[^ ^7^, ^8^ ^]^ Research has indicated that IDD has a significant genetic susceptibility.^[^ ^9^ ^]^ Therefore, targeting the regulation of coding or coding genes linked to IDD based on gene therapy holds great promise as an approach for IDD repair. However, traditional gene therapy relies on viruses or elemental nano‐biological particles as gene carriers, with drawbacks such as repetitive injections, temporary medication release, and threats to the host's immune system.^[^ ^10^ ^]^ Hydrogels, hydrogel microspheres, and nanofiber scaffolds are examples of regenerative materials with strong biocompatibility that have demonstrated some application promise in IDD healing,^[^ ^11^, ^12^ ^]^ alone or as medications and stem cell carriers. Therefore, introducing gene drugs or premodified gene drugs into regenerative materials and developing gene‐functionalized regenerative material (GRM) based on physical inclusion or formation of chemical bonds (amide bonds, Schiff base bonds, borate bonds, etc.) may be a new IDD repair strategy. Meanwhile, numerous investigations have demonstrated that GRM not only improves the local microenvironment and provides stress support for IDD but also realizes the root repair of the disease by targeting IDD‐related genes.^[^ ^13^, ^14^, ^15^, ^16^ ^]^ In the current review, the physiological and pathological characteristics of IVD, genetic susceptibility to IDD, and limitations of available treatment options were briefly explained, and then the significance of GRM‐based treatment of IVD and the challenges and future development direction in this field were prospected in the end (**Figure** **1**).

## 2 IVD Physiology and Pathophysiology

### 2.1 IVD Physiology

**Figure 2:**
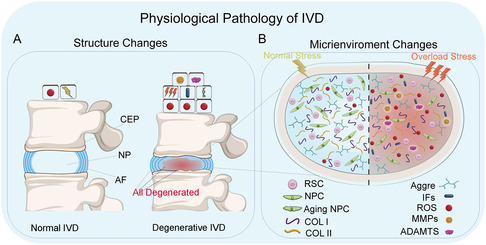
Disc pathophysiology: A) structural changes of IVD; B) microenvironment changes of IVD.

The IVD stands as the largest nonvascular organ within the human body,located between two neighboring vertebral bodies. The three components of the IVD are a flexible nucleus pulposus tissue, layers of fibrous annulus surrounding the nucleus pulposus, and a closed cartilage endplate^[^ ^1^, ^17^ ^]^ (**Figure** **2**A). The nucleus pulposus tissue contains proteolytic mucous matrix and nucleus pulposus cells (NPCs), which play the role of “ball” dispersing stress in IVD.^[^ ^4^, ^18^ ^]^ Besides, fresh nucleus pulposus has a high‐water abundance, which is closely related to the high‐water preservation capacity of proteoglycan. The abundant type II collagen fibers formed a loose three‐dimensional fiber network with NPCs, significantly enhanced the viscoelasticity of the nucleus pulposus, and provided a vital physiological basis for the resistance and dispersion of IVD stress.^[^ ^19^, ^20^ ^]^ The fibrous annulus, which tightly encircles the NP horizontally and carries on the final cartilaginous version longitudinally, comprises concentric collagen fiber flakes.^[^ ^21^ ^]^ The strong type I collagen fibers and the formed cross‐layer bridging network give the fibrous annulus good shear resistance and allow the IVD to rotate and bend in a particular range.^[^ ^21^, ^22^ ^]^ Furthermore, between the IVD and the vertebral body lies a layer of hyaline cartilage known as the cartilage endplate, and its internal content of collagen and proteoglycan directly determines its tensile properties.^[^ ^20^ ^]^ It performs an excellent mechanical support role, which can effectively disperse the high‐intensity pressure generated by the nucleus pulposus and protect the vertebra surface from damage.^[^ ^4^, ^23^ ^]^

### 2.2 IVD Pathophysiology

IDD has several different causes, the most common ones being aging, smoking, trauma, abnormal stress, and heredity.^[^ ^4^ ^]^ NPCs homeostasis imbalance, ECM degradation, and structural alterations to the IVD are the primary abnormalities associated with IDD (Figure 2B). NPCs homeostasis is the basis for maintaining IVD function.^[^ ^24^ ^]^ NPCs senescence, autophagy, and apoptosis are in dynamic balance to jointly support the homeostasis of IVD cells under physiological conditions.^[^ ^25^, ^26^ ^]^ Under pathological circumstances, however, NPCs homeostasis is destroyed, and the number and shape of NPCs change significantly.^[^ ^24^ ^]^ Overload stress and elevated reactive oxygen species (ROS) are common causes of NPCs homeostasis imbalance.^[^ ^27^, ^28^ ^]^ A specific amount of ROS regulates IVD signaling pathways as signaling molecules. In contrast, high ROS can cause premature aging and excessive apoptosis of NPCs.^[^ ^27^, ^28^ ^]^ In a similar vein, nucleus pulposus and annulus fibrosus homeostasis require specific stress activation. However, overload stress causes premature aging and autophagy failure of NPCs by upregulating oxidative stress levels and overactivating inflammatory pathways, thus accelerating disc progression.^[^ ^4^ ^]^ Collagen, proteoglycan, and water in the ECM are lost significantly during the IDD, and the collagen fiber arrangement in the annulus fibrosus becomes disorganized or even fractured.^[^ ^4^ ^]^ Osteophytes eventually grow, the nucleus pulposus protrudes, and the strength and stability of the IVD are significantly diminished. Common causes of ECM degeneration include NPCs homeostasis imbalance, inflammatory response, excess ROS, and overload stress. NPCs homeostasis imbalance leads to inadequate ECM synthesis, which in turn leads to IDD. In addition, the accumulation of inflammatory factors in the degraded IVD increased the content of matrix metalloenzyme (MMP) and accelerated the decomposition of ECM.^[^ ^29^, ^30^, ^31^, ^32^ ^]^

## 3 IVD Genomics

**Figure 3:**
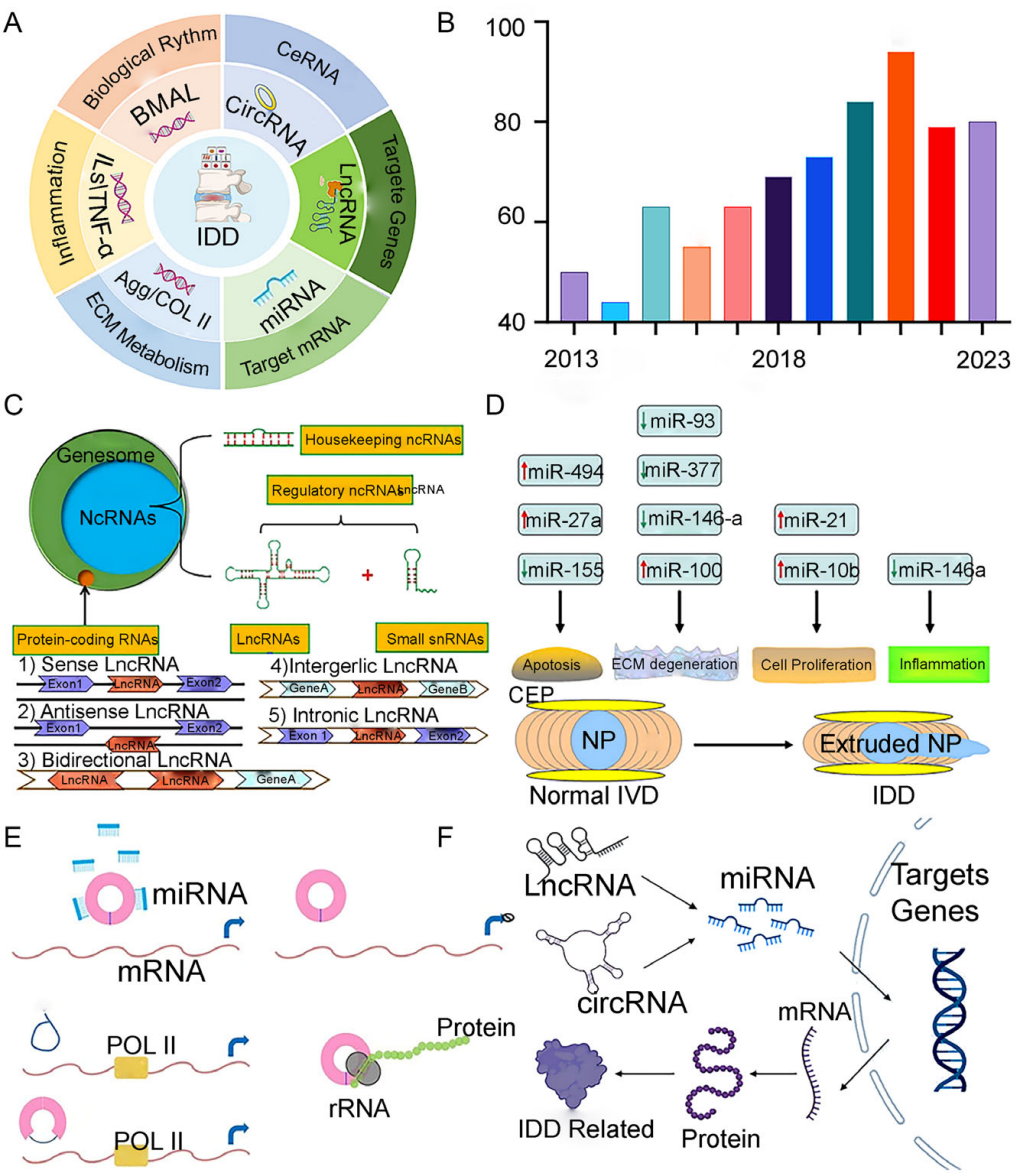
A) IVD‐related genes and their mechanism of action; B) the number of literature published in the past 10 years after inputting the keywords “IDD and Genetic susceptibility” in Web of Science database; C) schematic diagram of a common category of LncRNA; D) miRNAs are closely related to IDD development; E) schematic diagram of biological function of circRNA; F) noncoding RNA such as LncRNA, microRNA, and circRNA form crosstalk networks and play a role in regulating IDD. C–E) are reproduced with permission;^[^ ^47^, ^49^, ^50^ ^]^ F) is reproduced with permission,^[^ ^33^ ^]^ Copyright 2022, Frontiers.

The IDD has a genetic susceptibility, and susceptibility genes mainly include coding genes and noncoding genes^[^ ^9^, ^33^ ^]^ (**Figure** **3**A). We summarized the publications published in the last 10 years after entering “IDD and genetic susceptibility” into the Web of Science database for search purposes (Figure 3B).

### 3.1 IVD Degeneration‐Related Coding Genes

IDD‐related coding genes mainly encompass ECM metabolism, inflammation, and IVD clock genes (**Table** **1**). COL2A1 and ACAN genes are used to compose ECM for collagen and proteoglycan, respectively. Mutations or polymorphisms can impact type II collagen and glycan‐1 synthesis and function, which could affect the stability of IVD.^[^ ^34^, ^35^, ^36^ ^]^ MMP‐2, MMP‐9, and a disintegrin and metalloproteinase with thrombospondin motifs (ADAMTS) genes encode matrix metalloproteinase‐2, proteinase‐9, and thrombospondin motif‐containing disintegrin, respectively, and are crucial regulators of IVD ECM metabolism.^[^ ^29^ ^]^ The mutations or polymorphisms of these three genes are closely associated with IDD.^[^ ^4^, ^23^ ^]^ TIMP‐1 and TIMP‐2 are genes that encode inhibitors of matrix metalloproteinases, specifically inhibitor 1 and inhibitor 2, respectively. Variations in these two genes are linked to IDD degeneration and may impact the equilibrium between MMPs and the ECM of the IVD.^[^ ^37^, ^38^, ^39^ ^]^ Vitamin D receptor (VDR) gene polymorphism may be strongly associated with IDD. VDR gene encodes vitamin D receptor protein and controls the autophagy level of NPCs.^[^ ^40^, ^41^ ^]^ In addition, the genes encoding inflammatory factors are closely related to IDD‐related inflammation, matrix destruction, cell senescence, autophagy, apoptosis, and other pathological processes.^[^ ^42^, ^43^ ^]^ The central genes of IVD clock control, ARNTL and CLOCK, encode clock proteins, and BMAL1, respectively. These proteins work together to regulate the biological rhythm of IVD tissues through interaction.^[^ ^44^, ^45^, ^46^ ^]^

**Table 1:** IDD‐related susceptibility genes

Regulatory targeta)	Coding genes	Noncoding genes
ECM metabolism	ADAMTS^[^ ^137^, ^138^ ^]^	miR‐106b‐5p,^[^ ^144^ ^]^ miR‐3d,^[^ ^145^ ^]^ miR‐193a‐3p,^[^ ^146^ ^]^ miR‐338‐3p,^[^ ^147^ ^]^ miR‐150,^[^ ^148^ ^]^ miR‐499a‐5p^[^ ^149^ ^]^
	VDR^[^ ^40^, ^41^ ^]^	
	Aggrecan^[^ ^139^, ^140^ ^]^	
	Collagen‐II^[^ ^35^, ^141^, ^142^ ^]^	
	MMPs^[^ ^29^, ^39^ ^]^	LncR‐00917,^[^ ^150^ ^]^ LncR‐HOTAIR,^[^ ^151^ ^]^ LncR‐OIP5‐AS1,^[^ ^152^ ^]^ miR‐93,^[^ ^153^ ^]^ miR‐137,^[^ ^154^ ^]^ miR‐874‐3p^[^ ^155^ ^]^
	TIMP^[^ ^37^ ^]^	miR‐199a‐5p,^[^ ^107^ ^]^ miR‐15a,^[^ ^156^ ^]^ miR‐217,^[^ ^157^ ^]^ Lnc00641,^[^ ^158^ ^]^ miR‐26a‐5p,^[^ ^159^ ^]^ circR‐GRB10,^[^ ^160^ ^]^ miR‐155,^[^ ^161^ ^]^ miR‐30d,^[^ ^145^ ^]^ circERCC,^[^ ^162^ ^]^ circGPATCH6L,^[^ ^163^ ^]^ circR‐CIDN,^[^ ^164^ ^]^ circSPG21,^[^ ^165^ ^]^ miR‐145,^[^ ^166^ ^]^ miR‐338‐3p,^[^ ^147^ ^]^ miR‐24‐3p,^[^ ^167^ ^]^ miR‐573^[^ ^168^ ^]^
Inflammation level	IL‐1β^[^ ^42^, ^143^ ^]^	
	TNF‐α^[^ ^143^ ^]^	
NPCs homeostasis	ARNTL^[^ ^78^ ^]^	
	CLOCK^[^ ^46^ ^]^	

a) ECM: Extracellular matrix; IL: interleukin; MiR:microRNA; TNF: Tumor necrosis factor; NPC: Nucleus pulposus cell; LncRNA:Long‐non‐coding RNA; circR: circluar RNA; ADAMTS: A Disintegrin and Metalloproteinase with Thrombospondin motifs; MMP: Metallomatrix enzyme; VDR: Vitamin D receptor; TIMP: Tissue inhibitor of metalloproteinase

### 3.2 IVD Degeneration‐Related Noncoding Genes

The three primary noncoding genes associated with IDD are circular RNA (circRNA), microRNA (miRNA), and long‐non‐coding RNA (LncRNA) (**Table** **1**).^[^ ^33^ ^]^ LncRNA, which is only 200–400 bp long and cannot code for proteins, can control cell behaviors such as NPCs proliferation, apoptosis, autophagy, and ECM metabolism by binding to hub genes and miRNA.^[^ ^47^, ^48^ ^]^ They can be separated into five classes based on their locations and traits: justice, antisense, bidirectional, intergenic, and intron^[^ ^47^ ^]^ (Figure 3C). MiRNA are single‐stranded RNA molecules that form a chain‐like structure, while circRNAs have a closed‐loop structure due to a back‐splicing event that connects the 3′ and 5′ ends of the RNA molecule. The differential expression of miRNAs and circRNAs are crucial factors in both the physiological and pathological processes of IDD^[^ ^49^, ^50^ ^]^ (Figure 3D,E). Specifically, LncRNA, MicroRNA, and CircRNA often form a crosstalk network that controls ECM metabolism, inflammation level, and NPCs homeostasis, hence regulating the occurrence and progression of IDD^[^ ^33^ ^]^ (Figure 3F).

## 4 Treatment Strategies and Limitations of Disc Degeneration

### 4.1 Classical Treatment of IDD

Two approaches are used in the traditional management of IVDs: conservative management and surgical management.^[^ ^3^ ^]^ Acetaminophen and ibuprofen are two essential oral analgesics that are part of a conservative treatment plan. Furthermore, patients with unbearable pain and intolerance to surgery may benefit from epidural and periroot corticosteroid injections, which provide short‐term but temporary pain relief.^[^ ^51^ ^]^ A robust body is essential to conservative care. To relax local muscle tissue, encourage the prolapsed nucleus pulposus to return, ease discomfort, and eventually relieve tension on nearby spinal neurons, physical therapy is a crucial component of conservative treatment.^[^ ^51^, ^52^ ^]^ Endoscopic degenerating IVD nucleus pulposus and lumbar fusion can help relieve some of the chronic low back pain caused by IDD. Nevertheless, surgical treatment may have problems such as easy recurrence, postoperative complications, and second surgery.^[^ ^3^ ^]^ In addition, as tissue engineering technology continues to advance, artificial disc replacement has achieved comparable repair outcomes as lumbar fusion in the short and medium term.^[^ ^53^ ^]^ However, there are issues like frequent replacement, and it is unclear how effective this technology will be in the long run.^[^ ^54^ ^]^ As a result, even while current conservative and surgical treatment approaches can somewhat slow the progression of IDD, they are still only palliative. They cannot reverse the IDD process or regenerate a degenerative IVD.^[^ ^3^ ^]^

### 4.2 Regenerative Therapy of IDD

**Figure 4:**
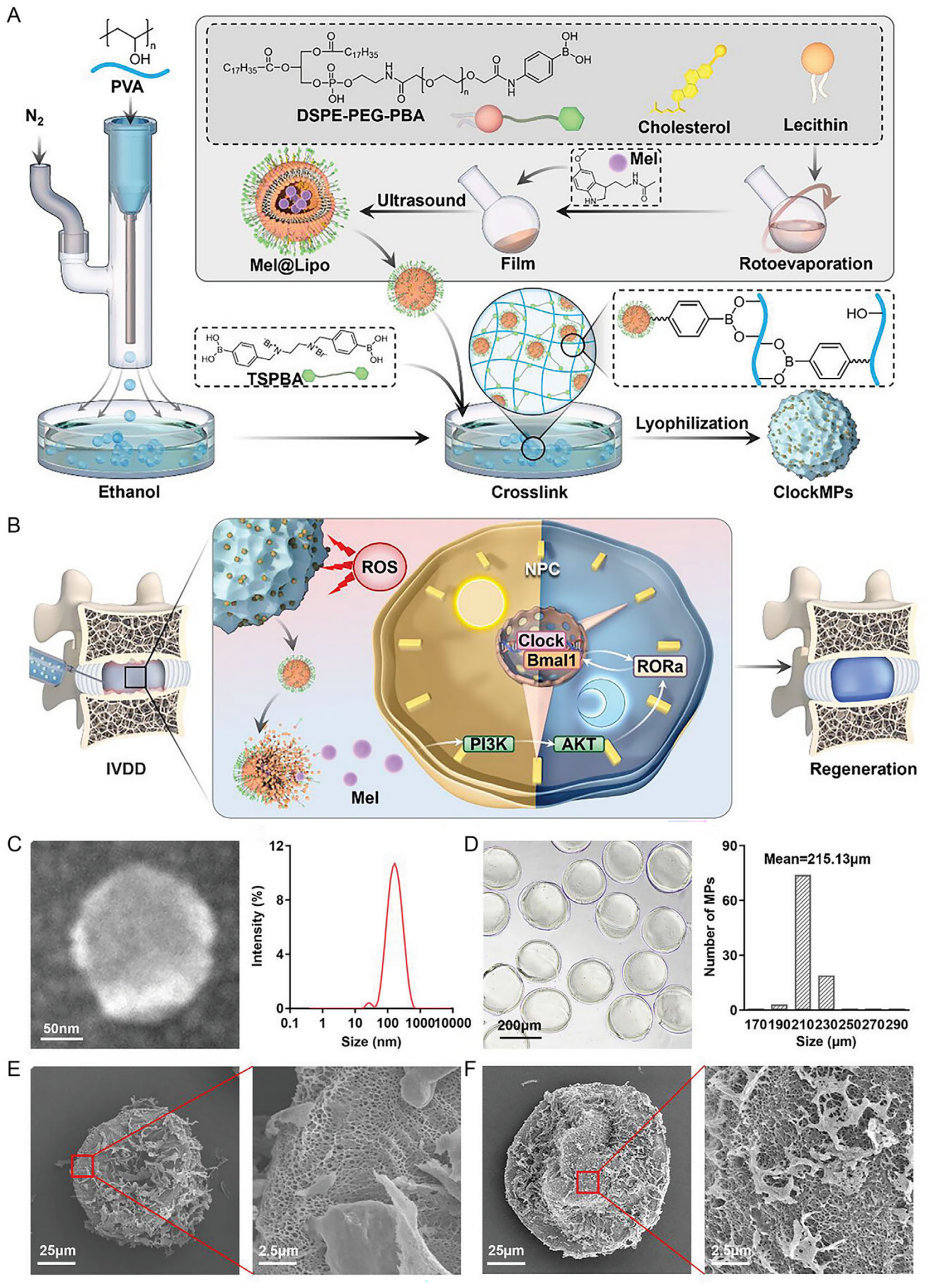
Small molecule drug functionalized regenerative materials promote disc regeneration. A) The diagram preparation of ClockMPs; B) ClockMPs reconstructs the IVD clock diagram; C) Mel@Lipo transmission electron microscope (TEM) image and particle size distribution; D)ClockMPs white light microscope image and particle size distribution; E) morphology of underwater gel microspheres (MP) under scanning electron microscopy (SEM); and F) ClockMPs configuration under SEM. A–F) are reproduced with permission,^[^ ^58^ ^]^ Copyright 2022, Wiley.

IDD repair based on disc pathophysiology has been extensively investigated based on regeneration materials, cells, genes, and simple bioactive medications (small molecule medicines, peptides, growth factors, and platelet‐rich plasma, among others).^[^ ^55^, ^56^ ^]^ Furthermore, more remarkable outcomes have been obtained when bioactive medications and cells were included in biomaterials for IVD repair.^[^ ^12^ ^]^ Much research has been done on using bioactive drugs (small molecules, protein polypeptide medicines, growth factors, etc.) to repair the IVD.^[^ ^12^, ^51^ ^]^ The poor internal microenvironment of degenerative IVD leads to dysregulation of IVD cell signal and abnormal function.^[^ ^4^ ^]^ Hydrogels and other regenerative materials to support bioactive and small molecule pharmaceuticals have increased drug use efficiency and improved disc repair effects.^[^ ^12^ ^]^ For instance, a ROS clearance scaffold containing rapamycin (Rapa@Gel) based on hydrogel‐supported rapamycin (Rapa) was created, which encouraged macrophage polarization toward M2 and reduced the degree of intradiscal inflammation by controlled drug release.^[^ ^57^ ^]^ In addition, Chen et al. prepared MT‐liposome nanoparticles (NPs) (MT@lip) based on liposome and melatonin (MT), loaded with microspheres prepared by polyvinyl alcohol, and prepared ROS‐responsive ClockMPs for reshaping the homeostasis of degenerative IVD clock^[^ ^58^ ^]^ (**Figure** **4**). Wu et al. inserted MT through bio‐mesoporous glass (MBG) and loaded it into alginate brine gel (SA@Gel) to prepare Mel‐MBG/SA@Gel. The findings of the experiments demonstrated that Mel‐MBG/SA@Gel with good stress tolerance may successfully control inflammation in the rat tail model, realize long‐term gradual release of MT, and be anticipated to promote disc regeneration further. This approach provided a new strategy for IVD repair that combines stress sharing and inflammation regulation.^[^ ^59^ ^]^ Zhu et al. constructed TGF‐β3/MnO2@NPs based on hollow manganese dioxide NPs (MnO2@NPs) loaded with TGF‐β3. According to experimental results, TGF‐β3/MnO2@NPs exhibited superior NPCs protection and ECM repair abilities in an oxidative stress environment compared to TGF‐β3 or MnO2@NPs alone.^[^ ^60^ ^]^

Stem cell delivery is a more direct IDD repair technique to improve IDD regeneration by enhancing the IVD microenvironment and promoting IVD cell viability, as opposed to bioactive medications such as small molecule pharmaceuticals, peptides, and growth factors.^[^ ^56^, ^61^ ^]^ In degenerative IVDs, the number of IVD cells decreases significantly, resulting in insufficient ECM synthesis and secretion, accelerating the IDD process.^[^ ^62^ ^]^ Therefore, the repair of IDD based on autologous or allogeneic stem cell delivery has been extensively studied, and fruitful research results have been produced.^[^ ^56^ ^]^ However, cell transplantation has the problem of low cell survival rate and biological activity.^[^ ^63^, ^64^, ^65^ ^]^ Research has indicated that the physical activity and survival rate of transplanted cells can be somewhat increased by pretreating them with hypoxia, chemical reagents, and a certain induction media.^[^ ^66^, ^67^, ^68^ ^]^ An injectable regenerative material with good biocompatibility is used as a cell scaffold to protect the transplanted cells’ biological function adequately.^[^ ^12^ ^]^ For example, hydrogels based on ultra‐pure alginate, hyaluronic acid (HA), methylcellulose, and gelatin NPs have been thoroughly researched for their ability to support mesenchymal stem cells (MSCs) and repair interdiscs.^[^ ^69^, ^70^ ^]^ Xu et al. prepared a gelatin methylacrylyl hydrogel into microspheres for loading growth differentiation factor 5 and MSCs and achieved good results in repairing IDD.^[^ ^71^ ^]^

In conclusion, based on the regenerated materials loaded with bioactive drugs and cells, the vitality of IVD cells is effectively increased by improving the IVD microenvironment, supplementing the number of IVD cells and providing stress support,^[^ ^12^ ^]^ etc., showing specific potential in IDD repair. Nevertheless, applying this regenerative therapy for disc repair still presents some difficulties: 1) adverse factors such as low pH, high level of inflammation, and IVD cells in the IDD are inhibited in their activity by oxidative stress, leading to a low uptake rate of cellular drugs; 2) the methods of pretreating the transplanted cells by biological, chemical, and physical means or enhancing the local microenvironment of the IVD with regenerative materials to increase the transplanted cells’ survival rate and activity are still questionable because the cells in vitro are relatively delicate; 3) autologous cell transplantation is invasive, which significantly reduces patient compliance. Apart from the invasive procedure of allogeneic cell transplantation, it is imperative to consider the transplanted cells’ immunogenicity; 4) the regenerative material has a specific elastic modulus, which is expected to achieve slow release of the drug when carrying bioactive drugs. Nonetheless, it is still a comforting measure rather than a cure, as it cannot stop the IDD process from the root.

### 4.3 Classical Gene Therapy of IVD Degeneration

Because IDD is genetically predisposed, controlling the disorder's pathogenic genes through gene therapy may be a valuable tactic for slowing the disease's progression.^[^ ^9^, ^72^ ^]^ Gene therapies such as messenger RNA (mRNA), plasmid (pDNA), small interfering RNA (siRNA), and (anti‐)miRNA (Anti‐miRNA/miRNA) are typically used to repair IDD.^[^ ^73^, ^74^ ^]^ Furthermore, gene editing technology (CRISPR‐Cas) has greater availability and potential to modify the genome with greater accuracy and effectiveness.^[^ ^75^ ^]^ In summary, many gene treatments with various therapeutic targets are available for IDD repair. It should be mentioned that a crucial component of gene therapy is transfection, and the effectiveness of transfection largely depends on the choice of gene vector. Currently, viral and nonviral gene vectors are utilized in IDD's conventional gene repair process.^[^ ^76^ ^]^

**Figure 5:**
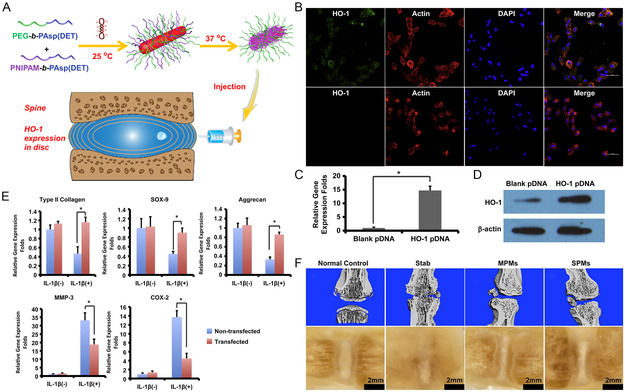
Thermal response hybrid bunched therapy loaded with heme oxygenase‐1 plasmid (HO‐1 pDNA) for IDD. A) Schematic diagram of HO‐1 pDNA preparation; B) representative confocal laser scanning microscopy (CLSM) images of transduced cells 48 h after transduction; C, D) the expression of HO‐1 in NPCs was also measured by real‐time fluorescence quantitative polymerase chain reaction (PCR) and western blot after transfection. The error line in the figure represents SEM, *n* = 4, *P < 0.05; E) real‐time fluorescence quantitative PCR analysis was performed to determine the expression of type II collagen, aggregator, SOX‐9, MMP‐3, and COX‐2 in 48 h NP cells after transfection. The error line in the figure represents SEM, *n* = 4, *P < 0.05; and F) representative micro‐computed tomography (μCT) reconstruction and rough appearance of rat tail spine at 4 weeks after in vivo gene therapy by the mixed polyplex micelles and Regular polyplex micelles prepared from sole block copolymer of PEG‐b‐PAsp loaded with HO‐1 pDNA. A–F) are reproduced with permission,^[^ ^87^ ^]^ Copyright 2019, Elsevier.

Viral vector has high transfection efficiency and is the first choice of traditional gene therapy for IDD. Nowadays, lentiviruses, hybrid baculovirus, adenoviruses, adeno‐associated viruses, and retroviruses are frequently employed for transfection.^[^ ^73^ ^]^ Lentivarios are excellent gene carriers with a high gene‐carrying capacity and biosafety, and they have been extensively used in the study and repair of IDD‐related mechanisms. Farhang et al. proved that modifying the lentivirus's DNA using CRISPR based on inflammatory receptors was feasible to repair IDD.^[^ ^77^ ^]^ Michal et al. demonstrated that clock transcription factor BMAL1 was a crucial modulator of IVD ECM homeostasis and cell fate.^[^ ^78^ ^]^ He also pretreated IVD cells based on lentiviruses to establish an autonomous biological clock in mouse and human IVD cells, and further research revealed that cytokines and age regulated the IVD biological clock. In addition, inflammation‐induced ECM degradation partially throws off IVD circadian clock homeostasis.^[^ ^46^ ^]^ The mechanism of action of Hif‐1 and HIPPO signaling pathways in IDD was also studied with the help of lentivirus gene vectors.^[^ ^79^, ^80^ ^]^ Hybrid baculoviruses, renowned for their exceptional transfection efficiency, have garnered significant attention in conjunction with CRISPR technology. Their versatility and efficacy have rendered them a subject of extensive investigation, owing to their potential in various fields of research and application.^[^ ^81^ ^]^ NPs have recently been employed as gene delivery vectors because of their simple preparation, easy modification, and good biocompatibility.^[^ ^82^ ^]^ NPs commonly used for IDD repair include inorganic NPs, cationic liposomes, and polymer micelles. NPs have been employed as gene delivery vectors in recent years because of their easy biocompatibility, straightforward manufacturing, and facile customization. Polymer micelles, cationic liposomes, and inorganic NPs are common NPs for IDD repair.^[^ ^82^ ^]^ By preventing the expression of the genes ADAMTS5 and Caspase 3, Banala et al. used liposome‐based double siRNA transfection to treat IDD.^[^ ^83^ ^]^ Polyethylene glycol (PEG)‐polyplex nanomicelles were used by Chang et al. to transport Runx1 mRNA, which aided in IDD repair.^[^ ^84^ ^]^ Lin et al. supplied cartilage anabolic transcription factor (Runt)mRNA to treat disc disorders based on polyethylene glycol‐polyamino acid block copolymer.^[^ ^85^ ^]^ Bryant et al. demonstrated that encapsulating microRNA in liposomes and delivering it for the treatment of IDD is a workable strategy.^[^ ^86^ ^]^ Feng et al. prepared a heat‐responsive hybrid bundle loaded with heme oxygenase‐1 plasmid (HO‐1 pDNA), which effectively inhibited the expression of inflammatory factors and promoted the repair of IDD^[^ ^87^ ^]^ (**Figure** **5**).

In conclusion, traditional gene therapy based on virus or cationic NPs as gene carriers has yielded fruitful IDD repair research results. However, there are still a lot of issues: 1) host immunity issues can arise from virus vector transfection; 2) catalytic NP gene transfection relies on a positive charge, but too much positive charge might harm cells; 3) the issue of temporary drug release affects both virus and cationic NP loaded genes; 4) since IVD is a stress‐sensitive organ, conventional gene therapy is unable to maintain degenerative IVD, which may significantly reduce the repair effect in vivo.

## 5 Repair of IDD with Plasmid‐Functionalized Regenerative Material

Simple gene delivery methods like siRNA and mRNA can readily impact biological activity.^[^ ^88^ ^]^ By building plasmid expression vectors that connect target genes and alter host cells, it is possible to accomplish gene cloning, expression, editing, and delivery. Generally speaking, plasmids can treat disease by overexpressing particular protein molecules. Furthermore, the siRNA sequence can be cloned by the plasmid and produced as a “short hairpin RNA” (shRNA) inside the plasmid vector.^[^ ^73^ ^]^ The shRNA plasmid expresses the nucleotide sequence after it enters the cell, creating an active “double‐stranded RNA” (sRNA), namely siRNA, which is subsequently incorporated into the silencing complex (RNA‐induced silencing complex (RISC)). The double strands of siRNA are separated, and only the more stable strand is incorporated into the silencing complex to prevent the expression of particular genes.^[^ ^89^ ^]^ Traditional gene therapy‐based viral and cationic NP‐supported plasmids or single plasmid transfection have successfully regulated IVD cell activity and ECM regeneration in vitro. However, issues remain, including slow gene‐drug release and inadequate in vivo repair efficacy.^[^ ^90^ ^]^ For IDD repair, building gene‐functionalized regenerated regenerative material and adding plasmids is preferable.

### 5.1 Plasmid‐Functionalized Regenerated Materials Silence Gene to Repair IDD

**Figure 6:**
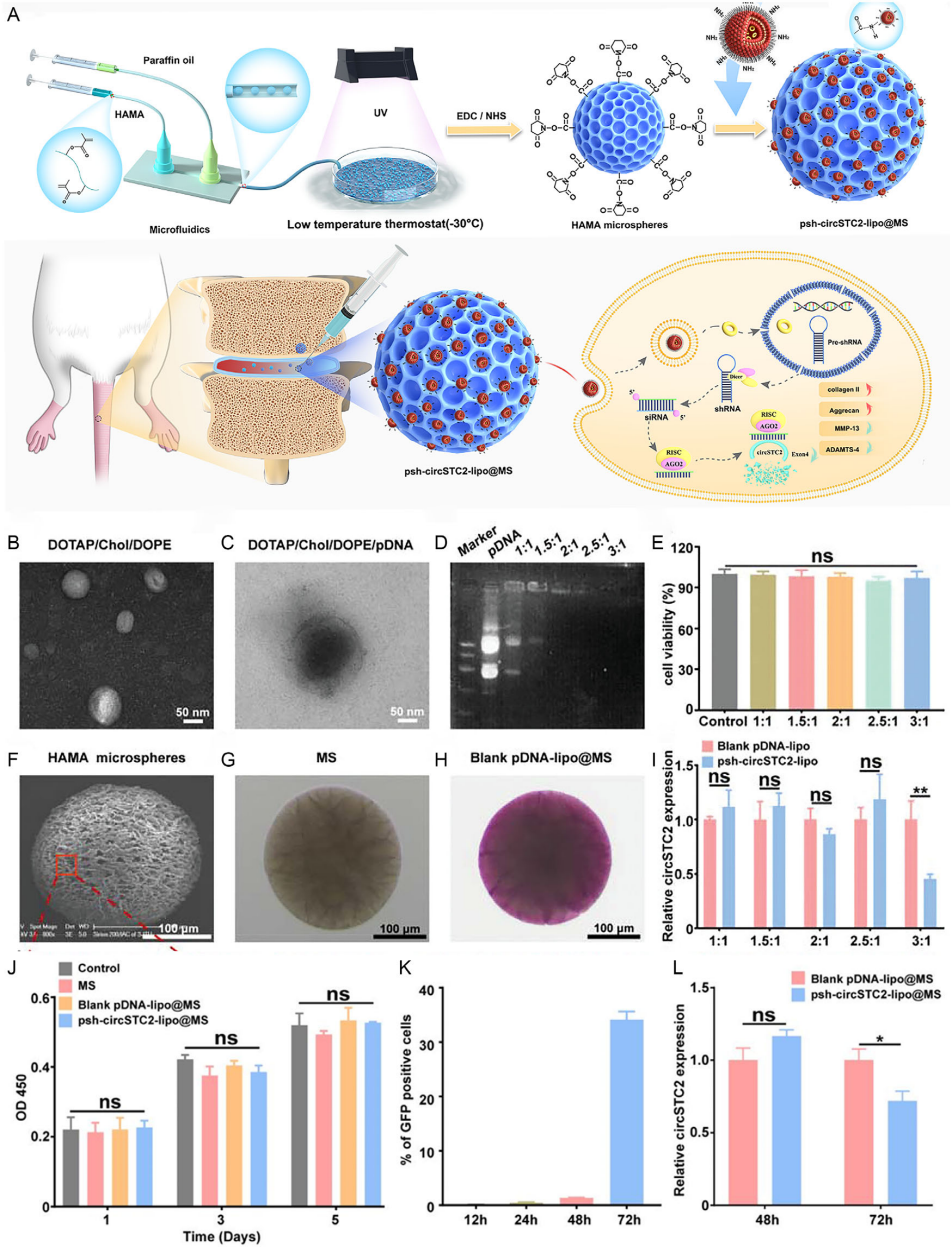
pDNA functionalized regenerative material for IDD repair. A) The diagram of circSTC72‐lipo@MS preparation and repairment for IDD; B, C) SEM and TEM images of DOTAP/Chol/DOPE liposomes; D) the pDNA binding capacity of DOTAP/Chol/DOPE liposomes at different N/P ratios was determined by AGAR gel electrophoresis; E) the effect of lipid complexes with different N/P ratios on the viability of NP cells; F) SEM images of HAMA microspheres; G–H) Dil‐stained microscope images and laser scanning confocal microscopy (LSCM) images; I) NPC transfection efficiency of lipid complexes with different N/P ratios; ns: not significant; **p < 0.01; J) CCK‐8 assays of NPCs after coincubation with MS, blank pDNA‐lipo@MS, and psh‐circSTC2‐lipo@MS for 1, 3, and 5 days; K) Flow cytometry detection of green fluorescent protein positivity rate after NP cells were transfected with Blank pDNA‐lipo@MS at different time points; L) qRT‐PCR detection of circSTC2 expression levels after coincubation of blank pDNA lipo@MS and psh‐circSTC2‐lipo@MS with NP cells for 48 and 72 h. ns: not significant; ∗*p* < 0.05. Reproduced with permission.^[^ ^13^ ^]^ Copyright 2021, Wiley.

Circular RNA is the noncoding RNA with a circular structure extensively present in cells. In general, circRNA can interact with miRNA as competing endogenous RNA, inhibiting the regulatory effects of miRNA on target genes and thus influencing pertinent signals. Based on bioinformatics analysis, Chang et al. constructed an in vitro model of human NPCs under nutritional restriction and discovered that the circSTC72 level was considerably upregulated.^[^ ^91^ ^]^ This implies that circSTC72 might help to facilitate IDD procedures. Consequently, silencing the circSTC72 gene might be a helpful strategy for disc relief. The biological activity of simple siRNA is unstable. However, the psh‐circSTC72 construct, made of siRNA sequences cloned in a plasmid vector as “short hairpin RNA” (shRNA), has increased stability. He prepared a cationic liposome, encapsulated pDNA based on electrostatic action, and designed a lipid complex (psh‐circSTC72@Lipo). Nevertheless, there are specific issues with leakage and temporary medication release with the single injection of a nanogenic vector for IVD degeneration. HA‐derived modified derivatives are widely employed in drug delivery because of their superior injectable and drug‐loading characteristics. Chang et al. used methyl propylene glycol (MA) and HA as raw ingredients to prepare injectable methacrylate esterified HA microspheres (HAMA@MS) for this investigation. It was discovered that HA and MA formed an ester bond in an alkaline environment and that there were several amino groups on the surface of psh‐circSTC72‐lipo. In this manner, coincubation created the amide link between PSH‐CircSTC2‐LIPO and HAMA, and psh‐circSTC72‐lipo@MS was ready to achieve gene functionalized of HAMA microspheres. According to experiments, psh‐circSTC72‐lipo@MS had good biocompatibility and slow release of gene drugs, which can be used to down‐regulate the expression of ECM‐decomposer enzymes like ADAMTS‐4 and MMP‐13, promote disc repair, and achieve long‐term silencing of the circSTC2 gene of NPCs in vivo and in vitro^[^ ^13^ ^]^ (**Figure** **6**). Therefore, plasmid functionalized regenerative material showed a better effect in IDD repair by silencing IDD‐related coding genes.

### 5.2 Plasmid‐Functionalized Regenerated Materials Overexpress Gene to Repair IDD

**Figure 7:**
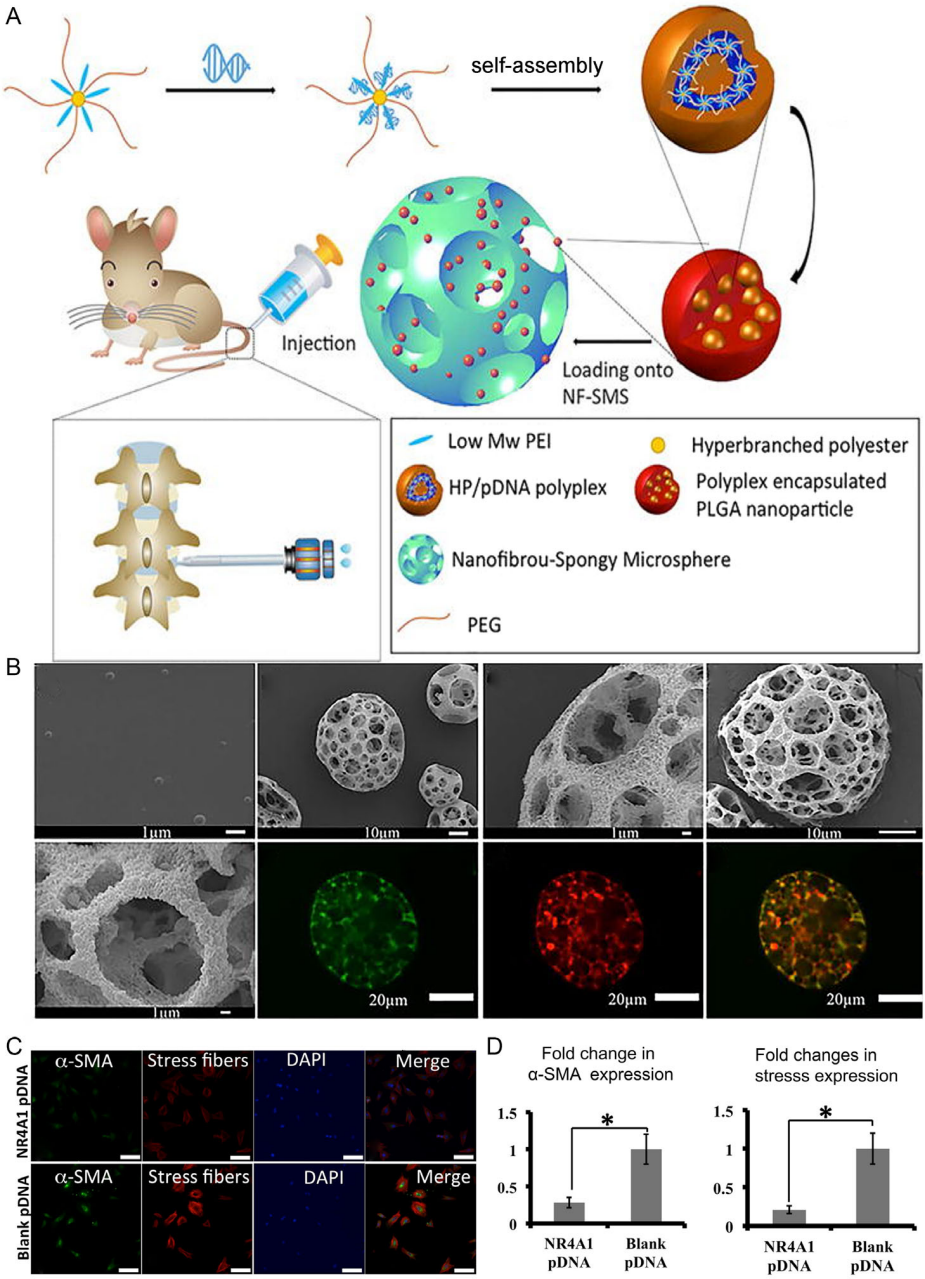
A) NR4A1‐HP@NS schematic diagram of IDD preparation and repair; B) NF‐SM images loaded with HP‐pDNA under SEM and confocal microscopy; C,D) Immunofluorescence staining for alpha‐smooth muscle actin and stress fibers was dramatically decreased in the NR4A1 pDNA transfected group (HP loaded with NR4A1 pDNA) compared to the blank pDNA group (HP loaded with blank plasmid DNA, which indicated that the fibrogenic effect of TGF‐β was suppressed by NR4A1 overexpression. A–D) are reproduced with permission,^[^ ^95^ ^]^ Copyright 2021, Elsevier.

IDD is commonly accompanied by irreversible ECM fibrosis.^[^ ^4^ ^]^ An important member of the nuclear transcription factor family, orphan nuclear receptor 4A1 (NR4A1) is expressed in a variety of cell types and tissues. It controls a wide range of physiological functions, including immunological response, energy metabolism, inflammatory response, and neurodevelopment.^[^ ^92^, ^93^ ^]^ The research discovered that by enlisting a blocking complex including SP1, SIN3A, LS1, and HAC1 into TGF‐β target genes, NR4A1 successfully reduced the profibrotic TGF‐β effect.^[^ ^94^ ^]^ Furthermore, a few small molecule NR4A1 agonists successfully prevented experimentally induced fibrosis in mice's kidneys, liver, skin, and lungs by stimulating NR4A1 activity.^[^ ^92^, ^93^, ^94^ ^]^ Consequently, Feng et al. hypothesized that NR4A1 overexpression might help prevent ECM fibrosis. He developed a hyperbranched polymer (HP) carrier in which a short PEG chain and a low molecular weight cation PEI were attached to the shell of the hyperbranched hydrophobic molecular core. To efficiently transfect pDNA, the HP vector can self‐assemble into nanopolymers with a “double shell” shape. These multistranded bodies were then encapsulated in biodegradable polylactic‐coglycolic acid (PLGA) nanospheres (NS), enabling two‐stage delivery of gene drugs. Lastly, pDNA‐NS was combined with recently created nanofiber sponge microspheres (NF‐SMS) in physical embedding to increase the sustained release effect of gene drugs. An injectable scaffold was made to realize gene functionalizedization of NF‐SMS, which was used for long‐acting sustained release of pDNA‐carrying multichains and effective transfection of pDNA. By local injection of pDNA‐NS@NF‐SMS, progressive transfection of NR4A1 inhibits the TGF‐β pathway by inducing TGF‐β to bind to SP1 and effectively inhibits ECM fibrosis. In vivo tests revealed that the gene‐functionalized biological scaffold effectively inhibited disc fibrosis and promoted disc repair^[^ ^95^ ^]^ (**Figure** **7**). In conclusion, the study's findings suggested that, given the physiology and pathophysiology of IVD, creating functionalized regenerative materials with overexpressed plasmids was a viable approach to repair IDD.

## 6 Repair of IDD with RNAi‐Functionalized Regenerative Material

A method of posttranscriptional gene silencing (i.e., mRNA level) is called RNA interference, or RNAi. Generally, RNAi inhibits mRNA through miRNA and siRNA.^[^ ^96^ ^]^ miRNA originates in the nucleus and is a standard short‐chain noncoding RNA that binds to an RISC to form a new silencing complex (miRISC). miRISC identifies and selectively targets particular RNA regions and downregulates gene expression through translation inhibition, mRNA cleavage, and deadenylation.^[^ ^97^ ^]^ Long double‐stranded RNA that is broken in cells is the source of siRNA.^[^ ^47^ ^]^ siRNA targets and precisely degrades complementary mRNAs to attach to RISC in the cytoplasm. In contrast to miRNA, siRNA is only utilized to silence a particular mRNA and is extremely specific and functionalizedly single since it is entirely complementary to mRNA.^[^ ^33^, ^89^ ^]^ In conclusion, RNAi composed of miRNA and siRNA is a helpful tactic to suppress gene transcription levels, and studies on IDD repair have produced some encouraging findings.^[^ ^89^, ^98^ ^]^ Excellent outcomes have been obtained by adding siRNA and miRNA to the regenerative material and creating RNAi‐functionalized regenerative materials for IDD repair.^[^ ^88^ ^]^

### 6.1 Repair of IDD with SiRNA‐Functionalized Regenerative Material

**Figure 8:**
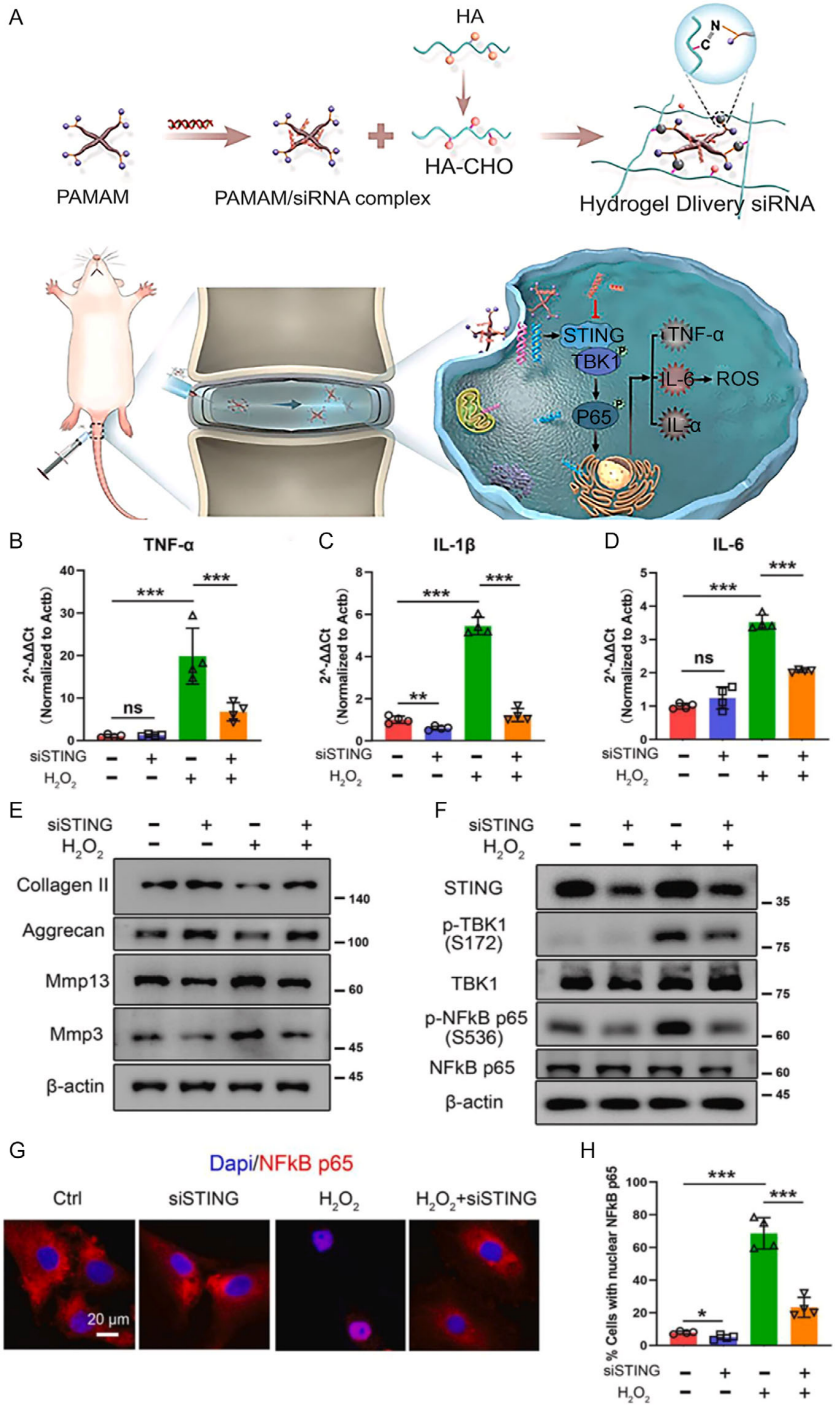
A) The synthesis and application diagram of siSTING@HP gel; B–D) NPCs were pre‐exposed to negative control siRNA or siSTING and then treated with 500 μM H_2_O_2_ for 48 h. The relative mRNA levels of tumor necrosis factor (TNF)‐α, interleukin (IL)‐1β, and IL‐6 were determined by qPCR (*n* = 4 independent experiments); E) representative western blot images of collagen II, aggregator, Mmp3, and Mmp13 proteins expressed in treated NPCs from three independent experiments. e.g. NPCs were pre‐exposed to negative control siRNA or siSTING, followed by 500 μM H_2_O_2_; F) representative western blot images of STING‐NF‐κB pathway expression in treated NP cells from three independent experiments; G) representative immunofluorescence (IF) staining of p65 in treated NP cells; H) quantitative analysis of cells with nuclear p65 in IF images (*n* = 4 independent experiments). The values shown are SD ± mean. *P < 0.05; **P < 0.01; *P* < 0.001; ns, no statistical significance. Statistical significance was assessed by one‐way analysis of variance and post‐hoc Tukey test. Reproduced with permission^[^ ^14^ ^]^ Copyright 2023, Elsevier.

The gene known as the stimulator of interferon (STING) regulates the immune system and is crucial to the inflammatory process. Research has revealed a considerable rise in the expression level of the STING gene in IDD.^[^ ^99^ ^]^ Chen et al. hypothesized that the STING gene overactivated signaling pathways linked to inflammation, thereby boosting the occurrence and progression of IDD. Thus, creating siRNA that targets the STING gene and achieving STING gene silencing may be an effective strategy for disc repair. However, endogenous ribonuclease readily breaks down the damaging naked siRNA molecules, making it difficult for them to get through the plasma membrane. A wide range of nanocarriers have been utilized for siRNA delivery to improve RNA stability and cell absorption due to the quick growth of nanotechnology. Even so, these systems still suffer from the typical rapid removal of nanocarriers. Significantly, more significant loading concentrations or more frequent dosages are necessary because of the short half‐life of siRNA, which can raise costs and increase toxicity. Consequently, Chen et al. created a PAMAM/siRNA nanogene complex using the electrostatic adsorption concept and positively charged polyamide dendrimer (PAMAM) to store STING‐siRNA. Chen et al. then prepared aldehyde‐functionalized HA (HA‐CHO) through classical oxidation. PAMAM/siRNA and HA‐CHO were comixed to prepare siRNA gene functionalized hydrogels by establishing dynamic Schiff base bonds (siSTING@HP). In conclusion, HA was chosen as an aldehyde‐functionalized polymer chain because of its exceptional biocompatibility. PAMAM functioned as an active crosslinking agent to create hydrogels and formed complexes with siRNA to promote siRNA transfection. The outcomes demonstrated that the PAMAM/siRNA complex, which has strong gene silencing effects, delayed the release through dissociation of the Schiff base bond. Lastly, by extending STING knockdown, siSTING@HP gel considerably reduced disc inflammation and reduced disc degeneration in a rat disc degeneration model caused by punctures^[^ ^14^ ^]^ (**Figure** **8**).

**Figure 9:**
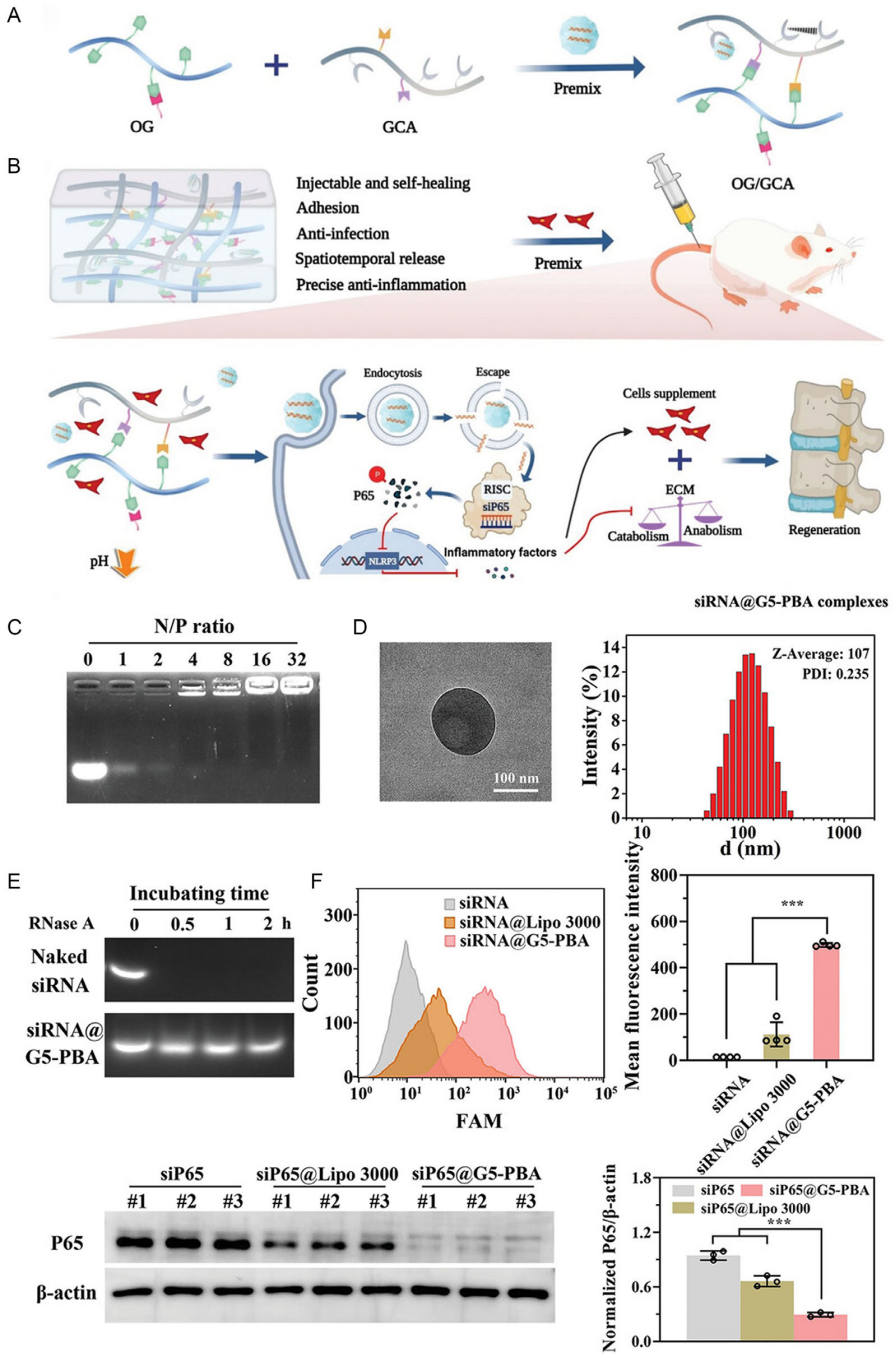
siRNA functionalized regenerative material for the treatment of IDD. A) The diagram of siRNA@G5‐PBA hydrogel preparation process; B) siRNA@G5‐PBA hydrogel properties and the mechanism for repairing IDD; C) siRNA@G5‐PBA agarose gel electrophoresis experiment of each group; D) optimal TEM image and size distribution of siRNA@G5‐PBA complex; E) RNase digestion at different time points, naked siRNA and siRNA@G5‐PBA agar‐agar gel experiments; F) the rat NPCs treated with siP65, siP65@lipo3000 and siP65@G5PBA were subjected to flow cytometry and WB assay, and the results were quantitatively analyzed. A–F) are reproduced with permission.^[^ ^101^ ^]^

An important function of the NF‐κB signaling pathway is to control the inflammatory response.^[^ ^100^ ^]^ p65, an NF‐κB component, is essential for inflammatory reactions. It was discovered that the activity of p65 was significantly enhanced in the degenerative disc.^[^ ^100^ ^]^ Inhibiting p65 levels may be a valuable strategy to reduce inflammation, heal the disc, and resolve other related issues based on P65‐siRNA. The point of siRNA delivery needs to be fixed. Cationic polymers modified with phenylboric acid (PBA) are potential gene carriers. PBA binds tightly to the cell surface and selectively interacts with heat shock and lysosomal membrane proteins, so it has a high capacity for lysosomal escape. In addition, covalent borate ester linkages with microenvironmental responsiveness can be created using the unique chemical structure of boric acid. Chen et al. initially created siRNA@G5‐PBA NPs using p65‐siRNA and the fifth‐generation PAMAM (G5‐PBA). Nevertheless, RNAi technology has several disadvantages as well, such as its short half‐life, aberrant distribution, and rapid silencing impact. Consequently, developing regenerated materials for the delayed release and maintenance of siRNA biological activity is extremely important. One type of naturally occurring polysaccharide that has strong biocompatibility and biodegradability is dextran. Besides, gelatin is an ECM derivative that promotes cell adhesion, proliferation, and remodeling. Using Girard reagent T‐modified oxyglucan (OG) and grafted catechol‐coupled gelatin (GCA) with dihydrazide adipate (AH) as raw materials, Chen et al. created a novel in situ multifunctionalized hydrogel (OG/GCA) rich in dynamic chemical bonds. siRNA@G5‐PBA NPs were added to the hydrogel system before OG and GCA crosslinking. The gene functionalized GCA hydrogel, SiRNA@G5‐PBA, was obtained by borate ester bonding between PBA and GCA. The results demonstrated that both in vivo and in vitro, the hydrogel could effectively control and maintain siRNA release over a 28‐day period. In addition, the hydrogel was rich in imine bond, borate bond, and acyl hydrazone bond, and the embedded siRNA@G5‐PBA exhibited the pH response release feature, which matched the microenvironment characteristic of the degenerative IVD. The IVD can be locally injected with the pH‐responsive OG/GCA hydrogel system and stably fixed to enable spatiotemporal regulated release of gene therapeutics and spatiotemporal regulation of cellular gene expression. Long‐term suppression of the P65/NLRP3 signaling pathway significantly reduced the inflammatory cascade and disc healing. Crucially, multifunctionalized hydrogel gene therapy significantly boosted disc regeneration in a rat disc model when paired with NPCs cotransplantation^[^ ^101^ ^]^ (**Figure** **9**). The construction of GRM based on siRNA showed excellent application value in IDD repair by long‐term inhibition of the post‐transcriptional level of pathological genes of IDD.

### 6.2 Repair of IDD with MiRNA‐Functionalized Regenerative Material

**Figure 10:**
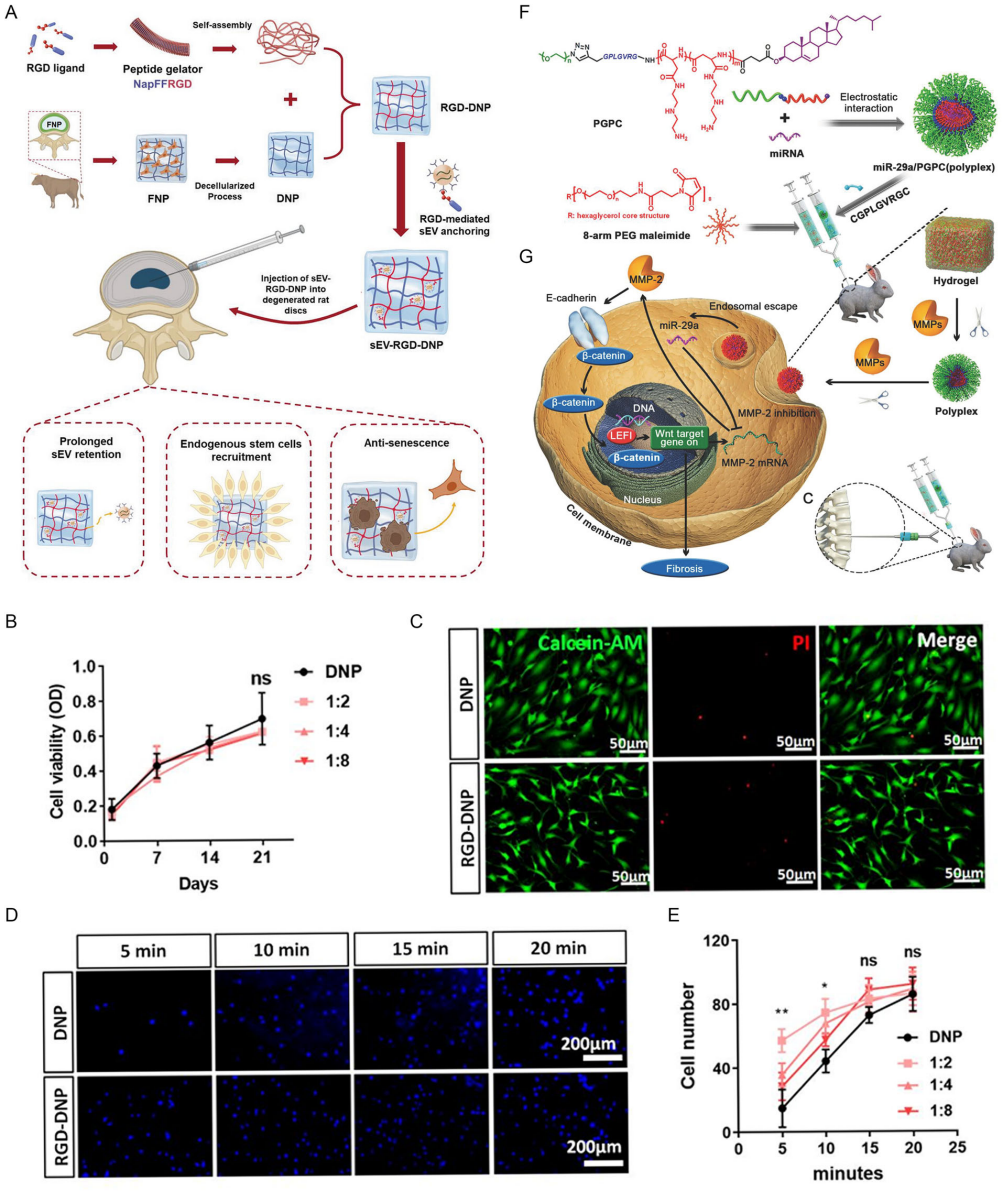
miRNA functionalized regenerative material for the treatment of IDD. A) Schematic diagram of exosomal mir‐3594‐5p functionalized hydrogel preventing disc degeneration by targeting the HIPK2/p53 pathway; B) cell viability detected by CCK8 assay indicated no significant difference between decellularized NP (DNP) and RGD–DNP hydrogels; C) Calcein‐AM and propidium iodide staining showed few cells death whenNucleus pulposus stem cells were plated on DNP and RGD‐DNP (1:2) for 21 days. Scale bar, 50 μm; D,E) Diamidino‐phenyl‐indole staining to evaluate the cell adhesive property of hydrogels revealed that RGD‐DNP (1:2) exhibited the best adhesive capacity on 5 min, whereas cell number was not different among groups on 20 min. Scale bar, 200 μm. Data are presented as the mean ± SD, *n* = 3. ns, nonsignificance. *p < 0.05, **p < 0.01 among groups. F,G) Schematic of the mechanism of sustained and bioreactive two‐stage delivery of therapeutic miRNAs by injectable hydrogels loaded with composite micelles to inhibit disc fibrosis. A–E) are reproduced with permission,^[^ ^102^ ^]^ Copyright 2023, Wiley. (G) and (F) are reproduced with permission;^[^ ^16^ ^]^ Copyright 2018, Wiley.

In degenerative IVD cells, homologous domain interaction protein kinase 2 (HIPK2) is overexpressed, which speeds up the IDD process by encouraging the production of aging genes like p16, p21, and p53.^[^ ^4^, ^9^ ^]^ As a result, it is anticipated that blocking HIPK2 will slow down the IDD process. The miR‐3594‐5p, abundant in extracellular vesicles (EVs) produced from stem cells, resists NPCs senescence. Nevertheless, EVs are susceptible to elimination by the microenvironment in the body, which significantly reduces the efficiency of their use. Peng et al. prepared arginine–glycine–aspartate (RGD) tripeptide complex core pulposus matrix hydrogel (RGD‐NP). By binding with surface integrin, EV was securely anchored inside the hydrogel, and gene functionalizedization of RGD‐NP hydrogel was realized. Based on preparation, sustained‐release EV released miR‐3594‐5p and inhibited HIPK2 protein expression by targeting HIPK2 messenger RNA, thereby suppressing the activation of p16, p21, and p53, delaying the premature aging of NPCs and repairing IDD^[^ ^102^ ^]^ (**Figure** **10**A,B).

The level of the metal matrix proteinase‐2 (MMP‐2) gene is markedly upregulated in degenerative IVDs, and this leads to an increase in ECM catabolism and an acceleration of the IDD process.^[^ ^29^ ^]^ The application of the microRNA‐29 (miR‐29) family in treating IVD degeneration is restricted since appropriate local delivery mechanisms are not yet available despite the family's significant capacity to control fibrosis and its potential inhibitory effect on inflammation levels. Feng et al. hypothesized that the delivery of miR‐29a could inhibit disc fibrosis and reverse disc progression and used MMP‐responsive cationic block copolymers (PGPC) for complexing miR‐29a and prepared miR‐29a/PGPC. Furthermore, following the physical encapsulation, the mixture of CGPLGVRGC and miR‐29a/PGPC polymicelles was combined with pre‐modified PEG, and the hydrogel could be quickly formed in an injectable way through the Michael addition reaction to achieve gene functionalizedization of the hydrogel. The hydrogel in the IDD exhibited responsive and sustained two‐stage miRNA delivery due to high concentrations of MMP. This included the first stage, where miR‐29a/PGPC was released through hydrogel degradation that was responsive to MMP, and the second stage, where MMP‐responsive micelle deregulation was enhanced to improve cell internalization into myeloid nuclei and facilitate effective endosomal escape. The experimental results showed that the hydrogel continued to release miR‐29a, effectively downregulated the level of MMP‐2, and inhibited IVD fibrosis^[^ ^16^ ^]^ (Figure 10C,D). In conclusion, miRNA has a wide range of sources and powerful functions, and miRNA‐functionalized regenerative material shows certain advantages in IDD repair by targeting and regulating the posttranscriptional levels of IDD pathological genes.

## 7 Repair of IDD with MiRNA Agomir‐Functionalized Regenerative Material

miRNA antagonists (Agomir) are segments of miRNA that have been altered by thiophoric acid, methylation, and cholesterol. Agomir is restricted from playing a role by strong competitive interaction with mature miRNA in vivo, which prevents miRNA from complementary pairing with its target gene mRNA.^[^ ^33^ ^]^ Specifically, Agomir is easily taken up by cells.^[^ ^103^ ^]^ Agomirs with varying miRNA structures can efficiently control target gene expression, aiding in treating many illnesses. Agmoir by itself for disc repair is still troublesome, though. For IVD repair, GRM based on Agomir is therefore quite possible.

### 7.1 Repair of IDD with Antagomir‐21‐Functionalized Regenerative Material

**Figure 11:**
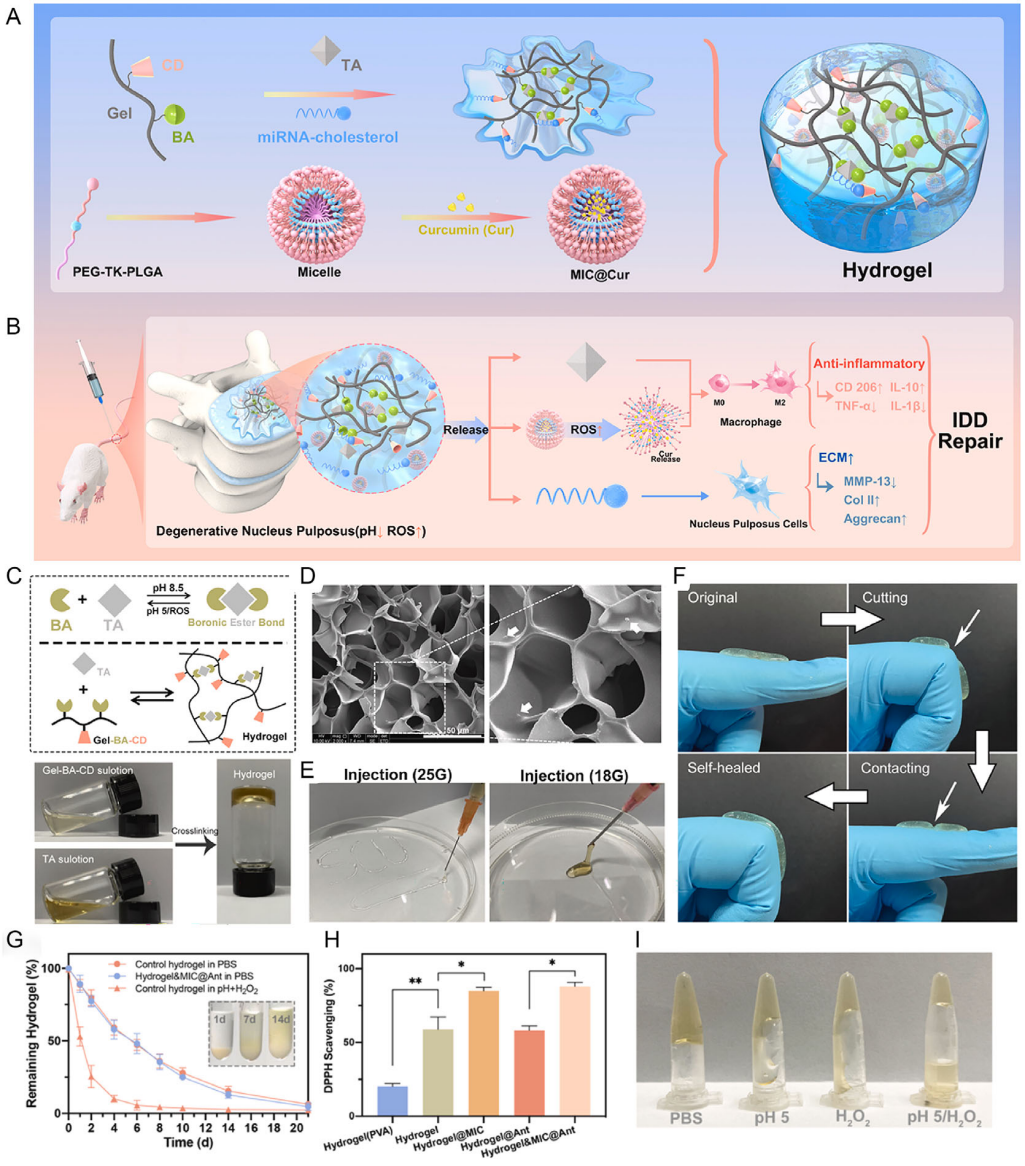
In the degenerative nucleus pulposus, sustained gene delivery via inflammation‐responsive anti‐inflammatory hydrogels supports the equilibrium of ECM metabolism. A) Schematic diagram of preparation of inflammatory hydrogel; B) Mechanism diagram of hydrogel repair of IDD. C) Preparation of hydrogels based on dynamic boron‐ester bond and gelation process thereof; D) porous microstructure of SEM underwater gel; E) pictures of glue produced by injection with different needles; F) pictures of the self‐healing process after hydrogel cutting. G) Results of in vitro degradation of underwater gels under different conditions; H) DPPH clearance percentage of hydrogels; I) Hydrogel images treated with PBS, HCl, H_2_O_2_, and HCl/H_2_O_2_. Data are presented as the mean ± SD, *n* = 3. ns, nonsignificance. *p < 0.05, **p < 0.01 among groups. A–I) are reproduced with permission,^[^ ^104^ ^]^ Copyright 2022, Elsevier.

The highly expressed miRNA‐21 in the IDD promoted the synthesis of MMP3, enhanced ECM catabolism, and expedited the IDD process. Therefore, targeted inhibition of miRNA‐21 may be an effective method to inhibit MMP3 levels and repair the IDD. Antagomir‐21 is an inhibitor of miRNA‐21 that has been modified by cholesterol and may enhance the ECM, yet its use in disc treatment has been restricted due to a suitable delivery mechanism. Phenylboric acid (BA) is a Lewis acid that forms reversible borate ester bonds by combining with *o*‐diol molecules. Tannic acid (TA) is a polyphenol obtained from plants that has particular anti‐inflammatory and antioxidant properties. In addition, many natural catechol/catechol groups exist in TA, which can combine with BA to generate boron ester linkages. Based on this, Wang et al. designed three different hydrogel delivery systems and realized gene‐functionalized hydrogels by physically embedding Antagomir‐21. β‐cyclodextrin (C) was grafted onto Gel‐BA (Gel‐BA‐C), and TA and Antagomir‐21 were added to create a hydrogel with anti‐inflammatory properties. Besides, curcumin, or Cur, has a solid inflammatory inhibitory effect, while excessive Cur might harm the body. Wang et al. created Mic@Cur based on ROS‐responsive amphiphilic polymer Micelle loaded Cur and integrated it into the premade hydrogel to accomplish the on‐demand release of Cur. According to the experimental findings, the IVD was repaired, and MMP3 expression was long‐actively inhibited by the targeted inhibition of miR‐21, which was accomplished by the long‐acting sustained release of Antagomir‐2^[^ ^104^ ^]^ (**Figure** **11**).

**Figure 12:**
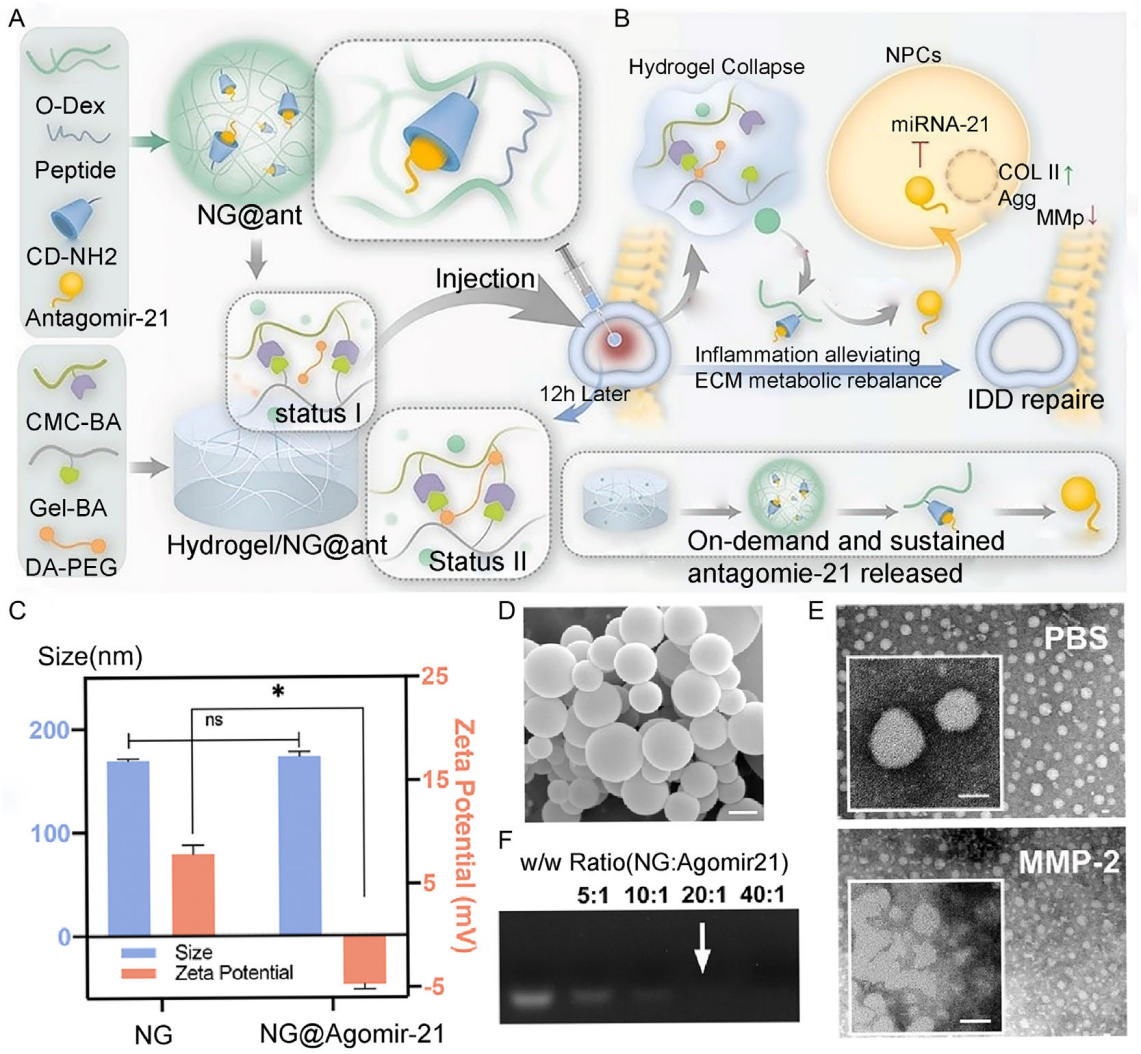
Gene‐polyphenol NPs functionalized hydrogel repair of IDD. A,B) self‐strengthening injectable nanogene hydrogel preparation and treatment of IDD; C) Dynamic Light Scattering (DLS) test NG and NG@Agomir‐21 particle size and potential distribution; D) Agomir‐21 images under SEM; E) NG@Agomir‐21TEM images after PBS and MMP‐2 processing; F) The stability of NG@Agomir‐21TEM was verified by agarose gel electrophoresis. A–F) are reproduced with permission,^[^ ^105^ ^]^ Copyright 2023, Elsevier.

“Self‐strengthening hydrogel” exhibits good injectability and excellent elastic modulus after injection. Based on the interaction between TA, miRNA, and cholesterol, Wang et al. encapsulated Antagomir21 in TA@NPs to generate TA@Antagomir21. After injection, “self‐strengthening hydrogel” exhibits an excellent elastic modulus and good injectability. TA@NPs were created by Wang et al. using an oxidative coupling reaction with TA in an aqueous solution. Antagomir21 is encased inside TA@NPs, and TA@Antagomir21 is produced based on the hydrogen bond between TA and the phosphoric acid skeleton of miRNA and the interaction between TA and cholesterol.^[^ ^15^ ^]^ Wang et al. also modified catechol/catechol groups and SH‐PEG‐BA on the surface of TA to prepare a reversible borate known as SH/TA NPs@Antagomir‐21. Next, they synthesized carboxymethyl cellulose (CMC)–GMA hydrogel with primary methacrylate instead of CMC. Antagomil gene functional hydrogel was prepared by Michal addition reaction after SH/TA NPs@Antagomir‐21 was introduced into hydrogel. Wang et al. then employed “click chemistry” to give the hydrogel improved properties following injection by using the prepared polyethylene glycol dipropionate (A‐PEG) as a crosslinking agent at the alkynyl terminal by means of propargylic acid and hydroxyl terminal PEG esterification in one step.^[^ ^15^ ^]^ In addition, Wang et al. prepared an inflammation‐responsive self‐strengthening hydrogel for miRNA delivery by physically encapsulating prefabricated NG@antagomir‐21 inside a premodified cyclodextrin. Such gene‐functionalized hydrogels are rich in emotional boron bonds, have low mechanical strength, and are easy to inject. After injection, spontaneous amino‐acetylene clattering polymerization made it self‐strengthening, prevented leakage, and provided specific principal support for IDDs. In summary, this gene‐functionalized hydrogel delivered antagomir‐21 into NPCs through three stages: regulated delivery of Nanogel@antagomir‐21 (NG@antagomir‐21) in an inflammation‐triggered hydrogel, NG breakdown after MMP‐2 specific cleavage of peptide bonds, and reversible physical action. The experiment results demonstrated that long‐term suppression of miRNA‐21 and MMP3 levels by the Antagomir‐21 gene functionalized hydrogel successfully improved IDD repair^[^ ^105^ ^]^ (**Figure** **12**).

### 7.2 Repair of IDD with Agomir874‐ and Anti‐miR‐199a‐Functionalized Regenerative Material

**Figure 13:**
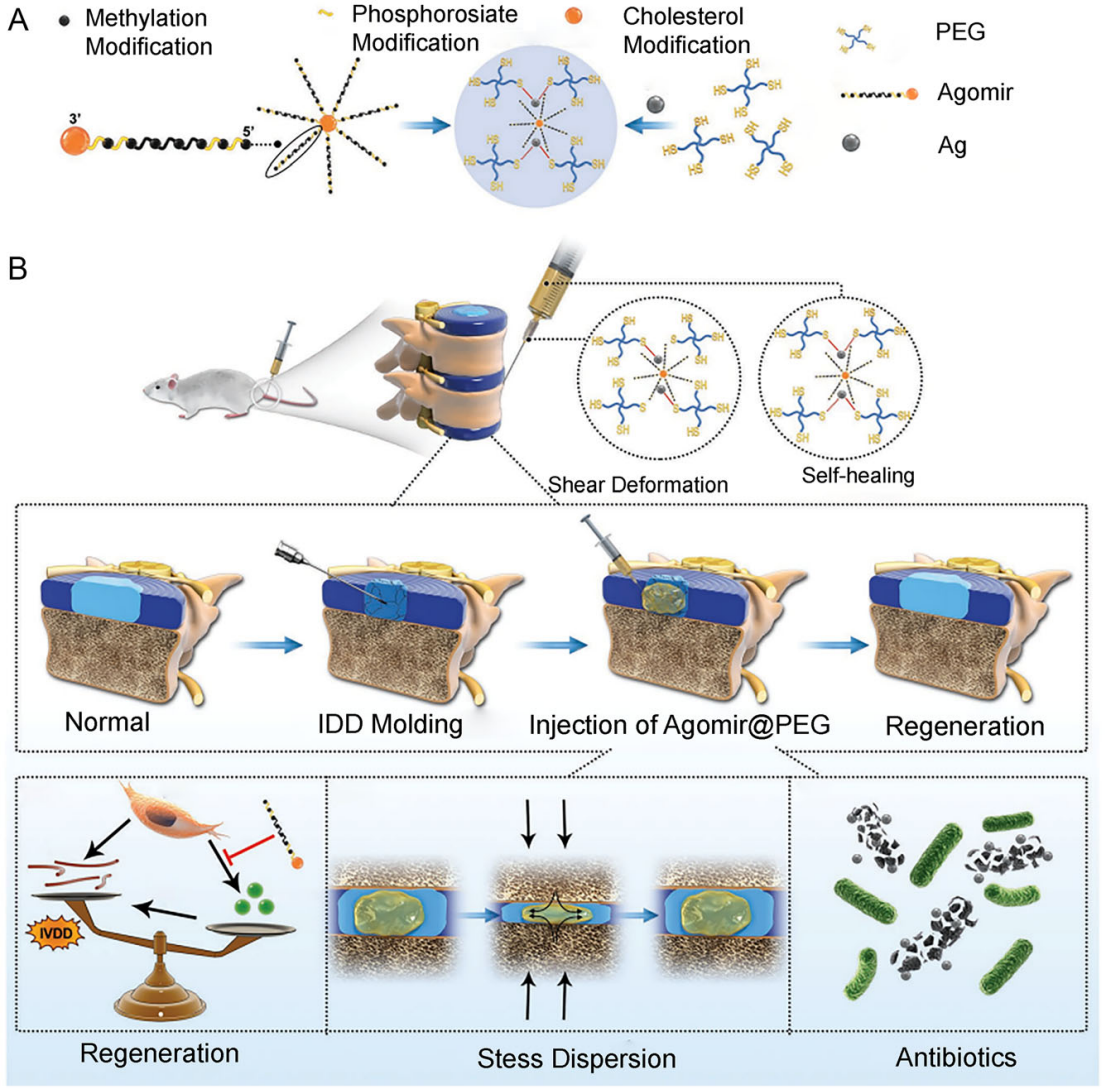
The nucleus pulposus's ECM metabolic balance was controlled by the gene‐hydrogel microenvironment. A) Preparation diagram of gene hydrogel; B) diagram of disc repair by gene hydrogel; A,B) are reproduced with permission.^[^ ^106^ ^]^

As mentioned earlier, MMP in the degenerative IDD is abnormally elevated, and ECM decomposes excessively, accelerating the IDD process.^[^ ^4^ ^]^ MiRNA‐874 was discovered to have the same function as miRNA‐21, promoting MMP synthesis. Consequently, Chen et al. believed that synthesizing Agomir874, which mimicked miRNA874, to downregulate MMP expression may be a strategy for disc repair. He therefore created a PEG hydrogel using the silver ion solution and Ag‐SH coordination of four‐arm PEG‐SH. By embedding Agomir874 in PEG hydrogel, the Agomir874 gene‐functionalized PEG hydrogel was created. The spontaneous synthesis of coordination bonds between Ag‐SH gelates PEG‐Ag hydrogels. The bond broke reversibly under shear forces, and the hydrogel transformed into an injectable fluid state. Simultaneously, hydrogels can achieve in situ self‐healing properties, providing favorable conditions for the release of genes and drugs. Consequently, the gene‐functionalized hydrogel effectively inhibited the expression of MMP and repaired IDD by improving the local microenvironment and realizing the long‐term slow release of Agomir874^[^ ^106^ ^]^ (**Figure** **13**). Disc degeneration is often accompanied by disc calcification, impairing the discs’ nutrient supply and metabolism, suppressing stem cell activity, and quickening the IDD process. As one of the essential transcription factors in NPCs, hypoxia‐inducing factor‐1 α (Hif‐1α) may help prevent disc calcification,^[^ ^79^ ^]^ and the overexpression of miR‐199a suppresses Hif‐1α production. Therefore, constructing miR‐199a antagonist, Antagomir‐199a, to upregulate the expression of HIF‐1α may be an effective strategy to inhibit disc calcification. Feng et al. synthesized HP based on hyperbranched double MPA polyester, polyethylene glycol, and polyethylenimide and packaged Antagomir‐199a based on charge anisotropy to prepare HP‐Antagomir‐199a multichain. Subsequently, the HP‐Antagomir‐199a multichains were physically embedded into PLGA NS and attached to NF‐SMS. Experimental results revealed that HP‐Antagomir‐199a@PLGA@NF‐SMS gene delivery system delivered Antagomir‐199a through hierarchical delivery and realized upregulation of Hif‐1α by blocking miR‐199a, effectively inhibiting disc calcification and repairing disc.^[^ ^107^ ^]^ In conclusion, the Agomir‐based gene‐functionalized nanofiber microspheres strongly affect IDD repair by competitively suppressing the corresponding miRNA.

## 8 Conclusion and Prospects

**Figure 14:**
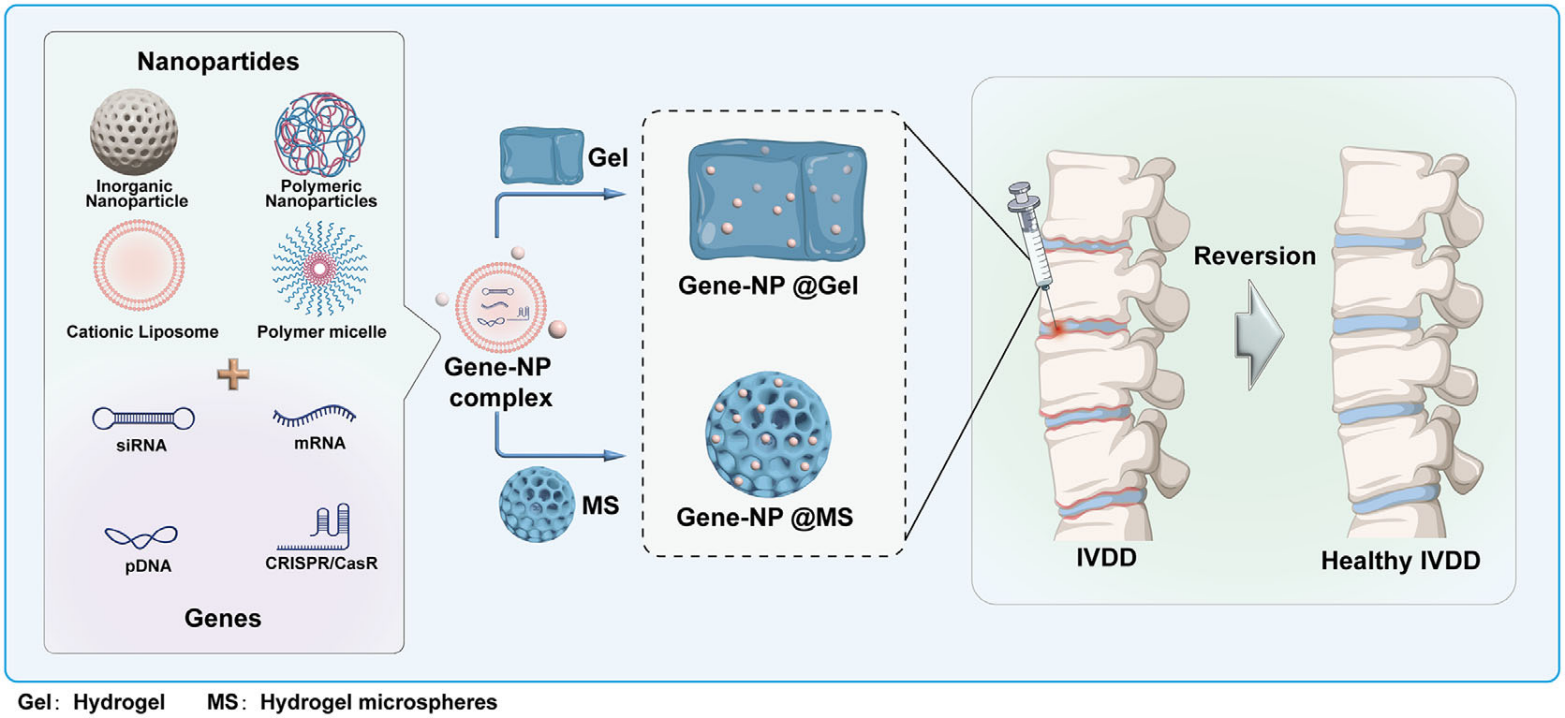
Schematic diagram of preparation of GRM and its application in disc repair.

IDD is a significant pathogenic component contributing to persistent low back pain, which severely lowers patients’ life satisfaction index and causes them to suffer from mental anguish and financial hardship.^[^ ^2^ ^]^ Disc degeneration cannot be repaired with the current restricted and inadequate therapy approaches for IDD.^[^ ^51^ ^]^ Although stem cells and bioactive medications loaded in regenerative materials have demonstrated some promise in aiding in the healing of degenerative IDDs, they can still not reverse the IDD process from the root.^[^ ^56^ ^]^ Conventional gene therapy, which relies on transfecting viruses or NPs, has drawbacks like temporary drug release and host immunity. It also cannot mitigate the damaging effects of aberrant stress on IVD cells.^[^ ^73^ ^]^ A workable method for IDD repair is to include naked or NP‐coated gene medicines into the regenerated materials and build GRM via physical embedding or chemical bond formation (**Figure** **14**).

Compared with traditional gene therapy, GRM achieves long‐term transfection of gene drugs, thus increasing the utilization efficiency and safety of gene drugs. In addition, the degenerative disc structure is unstable and unable to withstand daily stresses, which indirectly leads to the deterioration of the internal disc environment and accelerates the IDD process. Regenerated materials such as gene‐functionalized hydrogels, microspheres, and porous fiber scaffolds have good elastic modulus and can provide the necessary stress support for the degenerated IDD after in situ injection, which is highly advantageous for preserving IDD cell activity and enhancing the microenvironment of the IDD. GRM increases gene medication's safety and utilization efficiency by achieving long‐term transfection of gene pharmaceuticals, in contrast to standard gene therapy. Furthermore, the instability and inability to tolerate daily pressures of the degenerative disc structure cause the internal disc environment to deteriorate and speed up the IDD process. With their high elastic modulus, GRM like hydrogels, microspheres, and porous fiber scaffolds can give the degenerating IDD the stress support it needs following in situ injection, which is highly advantageous for preserving IDD cell activity and enhancing the microenvironment of the IDD. In summary, GRM can treat IDD by effectively enhancing the degenerative disc's local microenvironment and implementing root treatment of the disc. However, we must also look at the shortcomings of GRM for the treatment of IDD based on a comprehensive and developmental perspective.

### 8.1 Tissue Target

GRM's IDD gene therapy research focuses exclusively on the nucleus pulposus tissue due to the injection technique and cannot reach the cartilage endplate or the annulus fibrosus. In the future, synchronous gene therapy of annulus fibrosus, cartilage endplate, and nucleus pulposus can be accomplished through local minimally invasive puncture administration of fibrosus and cartilage endplate, in conjunction with emerging technologies like the ST needle drug delivery technology developed by Liu et al.,^[^ ^108^ ^]^ which may promote a more effective strategy for disc repair.

### 8.2 Cell Target

Currently, GRM primarily target the pathological genes of NPCs in IDD. However, IDD is a dynamic process involving inflammatory cells, particularly macrophages, which play a significant role in its development. Therefore, future advancements in GRM design should focus on accurately capturing biosignals within the degenerative disc microenvironment and reprogramming immune cells.^[^ ^109^, ^110^, ^111^ ^]^ This approach holds the potential to achieve more effective IDD repair outcomes.

### 8.3 Mechanism Target

Currently, encouraging ECM regeneration, reducing inflammation, and preventing IDD fibrosis are the main goals of GRM treatment for IDD. Nonetheless, the deterioration of the IVD clock, imbalance in mitochondrial rhythm, and aging of NPCs all contribute significantly to IDD development. Therefore, the selection of suitable regenerative materials based on bioinformatics analysis, the discovery of the above disc‐related pathogenic gene targets, and the development of feasible GRM can be used as the future research focus to support IDD repair.^[^ ^112^ ^]^

### 8.4 Single Target

Gene therapy has a high degree of precision but a narrow range. Disc degeneration is a pathological process regulated by several genes, mediated by several complex signaling pathways, and caused by numerous complex stimuli. Nevertheless, studies on GRM design based on the local microenvironment features of degenerative IDDs are scarce. Thus, multitarget therapy may be a hot topic in future research regarding gene therapy, given the current issues like ROS excess, inflammatory factor accumulation, and PH reduction.^[^ ^113^ ^]^

### 8.5 Biological Stress Microenvironment

Gene drugs have a gradual release effect thanks to GRM, which also prevents cytotoxicity from transitory gene‐drug release, lessens the further harm that aberrant stress causes to the degenerative IDD and creates an environment that is favorable to IDD regeneration. However, due to the harsh and delicate IDD microenvironment, future biological‐stress material designs must consider elasticity, viscosity, and biocompatibility.^[^ ^114^ ^]^ Intelligent vascularization 3D/4D/5D/6D printed tissue scaffolds have demonstrated significant advancements in tissue repair by combining precise regulation of mechanical structure and biological characteristics with vascularization.^[^ ^115^ ^]^ Moreover, Zheng et al. built functional microspheres (PFFM) based on microfluidic technology, and combined with 3D printing technology, placed them in polycaprolactone (PCL) 3D printing brackets. It is used to effectively promote rapid intervertebral fusion. It provides new ideas and alternatives for interbody fusion and other bone defects that are difficult to repair.^[^ ^116^ ^]^ Hence, the integration of 3D printing technology presents an opportunity to establish a comprehensive set of performance reference indicators specifically tailored for IDD maintenance using GRM. This endeavor would play a pivotal role in standardizing GRM‐based IDD maintenance approaches, ensuring consistent and reliable outcomes in the field.^[^ ^117^ ^]^

### 8.6 Gene Editing

GRM is now restricted to gene medicines, including pDNA, siRNA, and miRNA. The novel gene editing technology has shown promising therapeutic results in other domains and allows for the exact manipulation and editing of DNA sequences. The CRISPR/Cas9 system has emerged as a robust platform for mammalian genome engineering, enabling precise manipulation of genetic sequences.^[^ ^118^ ^]^ Over the past decade, significant advancements have been reported in the application of the CRISPR/Cas9 technology for the amelioration of IDD.^[^ ^77^, ^119^, ^120^ ^]^ Notably, the targeted ablation of β‐catenin signaling pathways, exemplified by the functional disruption of the FAN domain, has demonstrated a discernible attenuation of the degenerative process in IDD.^[^ ^121^ ^]^ Therefore, closely merging state‐of‐the‐art science and technology to produce CRISPER/Cas9 functionalized regenerative material may achieve more impressive IDD repair results, given the high genetic susceptibility to IDD.

### 8.7 mRNA Vaccine

RNA‐based therapeutics have catalyzed a revolutionary transformation in the biomedical field, offering unprecedented potential for disease prevention and treatment.^[^ ^122^ ^]^ In particular, mRNA as a novel class of therapeutic agents holds significant promise for the management and prevention of various diseases.^[^ ^123^, ^124^, ^125^, ^126^ ^]^ In recent years, the development of two highly efficacious mRNA vaccines by Moderna and Pfizer‐BioNTech, which have successfully mitigated the impact of COVID‐19, has underscored the immense potential of mRNA technology to revolutionize life sciences and medical research. Similarly, given the notable genetic predisposition associated with IDD,^[^ ^72^ ^]^ the prospect of developing mRNA vaccines with high efficacy for IDD prevention, drawing on the established methodologies of mRNA vaccine production, represents a pivotal direction for future research and development.

### 8.8 Drug‐Delivery Way

Deep within the body, IVD is prone to strain. Therefore, the current recommended method for treating IDD is a local injection, which satisfies the necessity for precision‐targeted medication delivery. However, patient compliance is significantly decreased by injectable administration, which is still intrusive and carries a particular risk of infection. Bioactive regenerated materials have shown promise in treating specific chronic bone and joint ailments orally in recent years.^[^ ^127^, ^128^, ^129^ ^]^ Thus, it may be a promising strategy to design GRM to target the regulation of patients’ overall metabolism or local IDD metabolism in the future. Furthermore, the utilization of microneedle patches for the administration of GRM in IDD treatment represents a valuable avenue of research due to its minimally invasive nature and long‐lasting effects. Developing such patches holds great potential for advancing IDD treatment strategies.^[^ ^130^ ^]^

### 8.9 Intelligent Responsiveness

Currently, scientists have created a range of microenvironment‐responsive regenerative materials based on the features of the degenerative disc's internal microenvironment, including low pH, accumulating ROS, and high expression of MMP.^[^ ^113^ ^]^ It must be noted that this class of drugs may not be able to achieve a genuinely long‐lasting microenvironmental response because of the disc's restricted volume. Hence, the development of GRM that is sensitive to both the intrinsic microenvironmental cues and extrinsic factors of IVD holds substantial practical significance. For instance, Zhang et al. have integrated black phosphorus nanosheets into the electrospinning process to fabricate a thermoresponsive multifunctional fiber scaffold with superior biomimetic characteristics, which has demonstrated promising outcomes in bone regeneration.^[^ ^131^ ^]^ Moreover, piezoelectric hydrogels have garnered extensive attention for their application in bone and wound healing due to their exceptional injectability, environmental sensitivity, and biodegradability. Notably, Wu et al. have modified ceramic hydroxyapatite and barium titanate using polydopamine and incorporated them into chitosan/gelatin matrices to create piezoelectric hydrogels with immunomodulatory, angiogenic, and osteogenic properties.^[^ ^132^ ^]^ Furthermore, Liu and Vinikoor have independently developed ultrasound‐responsive Atina hydrogels, which have effectively facilitated the repair of osteoarthritis, underscoring the potential of these materials in regenerative medicine.^[^ ^133^, ^134^ ^]^

### 8.10 Artificial Intelligence (AI)‐Assisted

In recent years, mRNA vaccines have been successfully commercialized and widely adopted.^[^ ^124^ ^]^ Compared to traditional vaccines, mRNA vaccines offer the advantage of being easily and rapidly updated to address viral mutations, thereby significantly reducing the time and cost associated with the development of new vaccines.^[^ ^125^ ^]^ However, the requirement for mRNA vaccines to be stored and transported at low temperatures imposes stringent conditions that limit their accessibility.^[^ ^135^ ^]^ To address this, Zhang et al. have utilized AI tools to optimize the sequence of mRNA vaccines, resulting in formulations with enhanced therapeutic efficacy and stability.^[^ ^126^ ^]^ Besides, Zhang et al. have developed an interdigitated (IP) sensor structure with a single‐layer parallel electrically coupled bilateral configuration, featuring only two electrodes, which meets the criteria for stable interaction and simplified wiring. By integrating the IP sensor into closed‐loop interactive entertainment systems, smart home systems, and user identification and authentication systems, they have demonstrated the sensor's multifunctionality. This integrated system highlights the potential of combining Internet of Things technology with AI.^[^ ^136^ ^]^ Therefore, the use of GRM to repair IDD while taking into account, noninvasive AI‐assisted rehabilitation therapy is a worthy direction of research.

### 8.11 Animal Models

The current animal model of IDD is restricted to small mammals with a significant capacity for self‐healing, which is undoubtedly different from the physiological and pathological characteristics of human IDD. Furthermore, unlike in animal models, human IDD is a chronic condition that might take decades to resolve. Animal models cannot bridge the years to decades it can take to evaluate long‐term therapeutic benefits. Therefore, based on animal including cattle, sheep, and monkeys, which have more similar physiological and pathological structures to human IDDs, the research data obtained may be more convincing by adopting a modeling method more suitable for IDD's pathogenesis.

## Conflict of Interest

The authors declare no conflict of interest.
